# Wavelength- and pH-Dependent Optical Properties of Aqueous Aerosol Particles Containing 4-Nitrocatechol

**DOI:** 10.1021/acsearthspacechem.4c00179

**Published:** 2024-11-12

**Authors:** Jamie W. Knight, Josephine E. M. Forsythe, Xu Zhang, Aidan Rafferty, Andrew J. Orr-Ewing, Michael I. Cotterell

**Affiliations:** † School of Chemistry, University of Bristol, Bristol BS8 1TS, U.K.; ‡ Department of Chemistry, University of Oxford, Oxford OX1 3QZ, U.K.

**Keywords:** brown carbon, single aerosol particle, cavity ring-down spectroscopy, complex refractive index

## Abstract

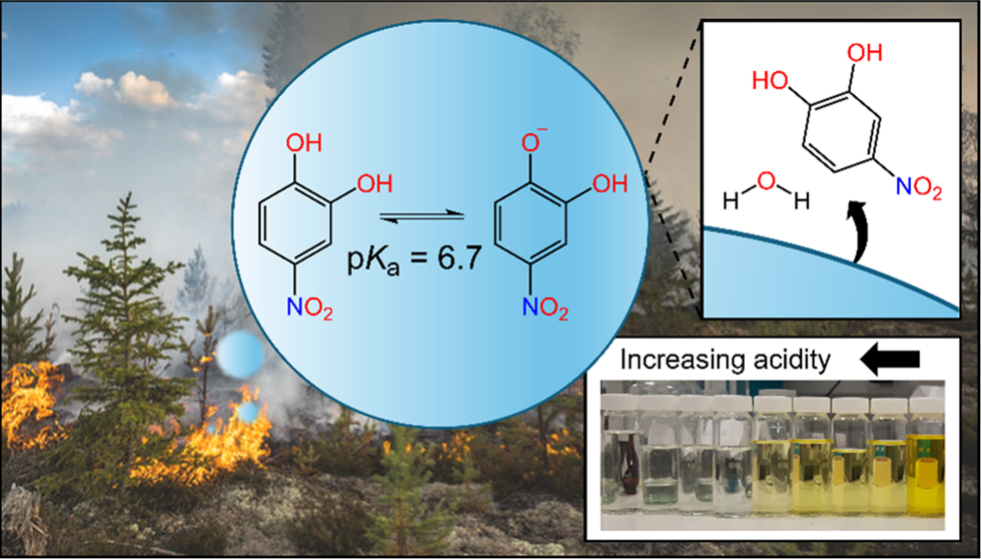

The radiative forcing caused by atmospheric aerosol represents one of the largest uncertainties in climate models. In part, these uncertainties derive from poor characterizations of the optical properties of light-absorbing brown carbon (BrC) containing aerosols. Here, single particle cavity ring-down spectroscopy (SP-CRDS) is used to determine the complex refractive index at the optical wavelength of 405 nm for aqueous particles composed of an abundant BrC species, 4-nitrocatechol. Moreover, the effect of acidity on the complex refractive index of 4-nitrocatechol is explored. UV/visible spectroscopy allows measurement of the wavelength-dependent (from 200 to 800 nm) imaginary refractive index for bulk aqueous solutions of 4-nitrocatechol, for which the pH is adjusted between ∼1 and 13. Applying a physically based refractive index mixing rule, wavelength-dependent imaginary refractive index values are estimated for the fully protonated, singly deprotonated and doubly deprotonated forms of 4-nitrocatechol. A Kramers–Kronig analysis constrained by the 405 nm SP-CRDS and 632.8 nm elastic light scattering measurements gives the wavelength-dependent real refractive index values. The real and imaginary refractive indices are essential for computing the radiative properties of these abundant BrC chromophores in aerosol plumes and cloudwater.

## 1 Introduction

Aerosols affect the radiative balance of the atmosphere directly by scattering and absorbing solar and terrestrial radiation, known as the direct aerosol effect. Aerosol particles that predominantly scatter sunlight back to space lead to cooling of the Earth’s surface. Conversely, tropospheric aerosol particles that are light-absorbing in the solar spectrum may have a net warming effect, depending on the relative magnitudes of the amount of light scattered and absorbed, and the albedo of the underlying surface. Black carbon (BC), a carbonaceous product of incomplete combustion that is strongly light-absorbing, is considered the largest contributor to total atmospheric aerosol absorption of visible sunlight.^1^ Nonetheless, the contribution from aerosol particles composed of light-absorbing organic carbon species, commonly referred to as brown carbon (BrC) because they typically absorb strongly at short visible wavelengths (λ ∼ 400 nm) and comparatively weakly at long visible wavelengths (λ ∼ 500–800 nm), is appreciable.^2^ However, the large number of unique chemical species in BrC aerosol, the temporal variation in the physiochemical properties of these species (i.e., atmospheric aging),^3,4^ and the wavelength-dependence of their light absorption, make the radiative effect of BrC on climate uncertain. Estimates of the radiative forcing caused by the direct aerosol effect of BrC aerosol vary by a factor of ∼15 (0.04–0.57 W m^–2^).^5,6^ In contrast, the radiative effect of BC is better constrained, with model estimates varying by a factor ∼4 (0.17–0.78 W m^–2^).^5−8^ The direct radiative effect for BrC is estimated to be about 30% of that for BC on a global scale,^8^ with ∼24% of the combined black and brown carbon tropopause warming attributed to BrC.^9^

Light absorption by BrC is largely associated with aerosol emissions from biomass burning and other combustion events.^10,11^ The incidence of biomass burning events (e.g., wildfires) is expected to increase globally as a result of warmer, drier conditions caused by climate change.^12^ Nitroaromatic compounds have been identified as abundant BrC components that are emitted directly into the atmosphere during combustion,^13,14^ and are formed efficiently as secondary organic aerosol from volatile organic precursors in biomass smoke.^10,15^ These nitroaromatic compounds are known to contribute significantly to total atmospheric light absorption by BrC aerosol.^13,14,16−18^ For example, Bluvshtein et al. determined the molecular contributions to UV/visible light absorption for BrC chromophores in water-soluble organic aerosols collected during a biomass burning event in Israel, with over 50% of the total light absorption attributed to nitroaromatic compounds including 4-nitrophenol, 4-nitroguaiacol, 4-nitrosyringol, and 4-nitrocatechol.^16^ Liang et al. also observed nitrocatechol species in high concentrations in wildfire plumes from Idaho and California.^19^ The authors found that these compounds partitioned predominantly (>90%) within aerosol particles, rather than as a gaseous species. Molecules in the aerosol particles interact with atmospheric oxidants less than those in the gas phase, suggesting that nitroaromatic compounds may be relatively long lasting in the atmosphere.

**Scheme 1:**
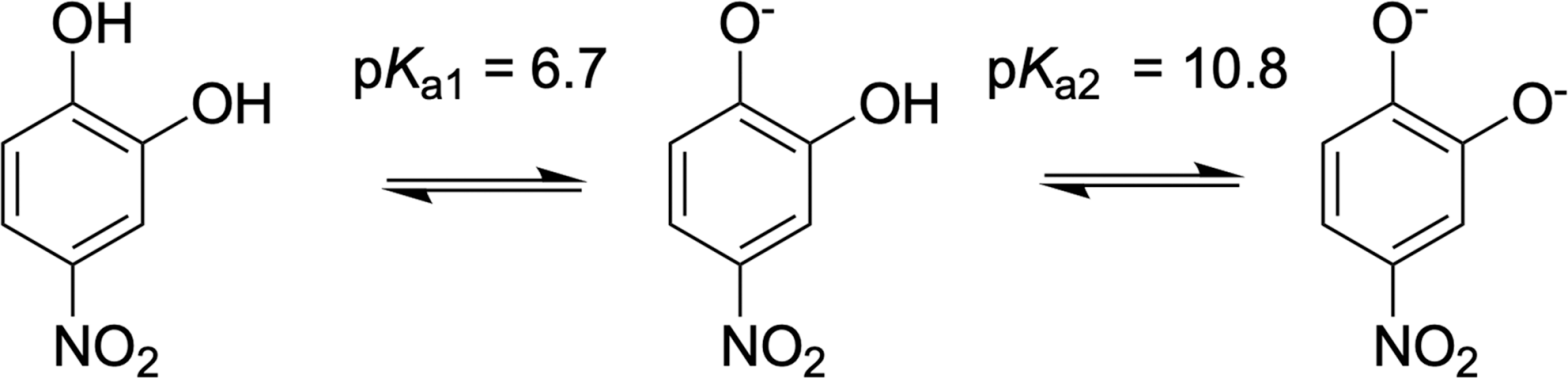
Schematic Diagram Showing the Structures of 4NC and Its Singly and Doubly Deprotonated Forms 4NC^–^ and 4NC^2–^ The p*K*_a_ values are taken from ref (23).

Zhao et al. have shown that the absorption bands across the visible spectrum for the weak acids 4-nitrophenol, 4-nitroguaiacol and 4-nitrocatechol are highly pH sensitive, with these band positions shifting to longer visible wavelengths with increasing pH.^20^ The p*K*_a_ values of fully protonated nitroaromatic compounds (∼4–7) are comparable to the pH of atmospheric aerosol (pH ∼ 1–5) and cloud droplets (pH ∼ 2–7).^21,22^ Therefore, the magnitude of the direct aerosol effect for particles containing these nitroaromatic species will depend on the particle acidity. Scheme 1 depicts the structures of the fully protonated, singly deprotonated, and doubly deprotonated forms of 4-nitrocatechol (4NC, 4NC^–^, and 4NC^2–^, respectively). The p*K*_a_ values for the formation of these deprotonated species, as determined by Cornard et al., are also shown.^23^ The fraction of 4-nitrocatechol that exists as 4NC^2–^ in the atmosphere is expected to be negligible, because the p*K*_a_ value for forming the doubly deprotonated species is well beyond the pH range accessed by typical atmospheric aerosol and cloud droplet populations.^23^

A key property that governs the optical properties and radiative forcing of BrC aerosol is the complex refractive index. The complex refractive index, *m* = *n* + *ik*, is closely connected to chemical composition and depends on the effective density, mean molecular weight and molecular polarizability of the particle.^24^ The real part, *n*, is a bulk property that quantifies the change in the speed of light through a medium. The imaginary component, *k*, describes the attenuation of light inside a medium and it is controlled by the concentration and absorption strength of any constituent chromophores. Price et al. measured the wavelength-dependence of the real and imaginary components of the complex refractive index for single, levitated aqueous particles containing 4-nitrocatechol by broadband light scattering.^25^ However, their measurements did not determine the particle pH or extend to wavelengths below 420 nm where 4NC absorption is strongest. The uncertainties in the imaginary component of the refractive index reached values up to ∼0.03 (±50%; see Figure S11 of ref (25)).

Using cavity ring-down spectroscopy (CRDS), we have recently demonstrated the measurement of the evolving extinction cross sections (σ_ext_) for single light-absorbing aerosol particles levitated in a linear electrodynamic quadrupole (LEQ) trap, as the particle size and chromophore concentration changed.^26−28^ The σ_ext_ is defined as the ratio of the power removed from an incident beam of light by an aerosol particle to the total incident irradiance. The particle size and composition dependent σ_ext_ values, and how they partition into scattering and absorption cross sections (σ_ext_ = σ_sca_ + σ_abs_), influence the magnitude of the direct aerosol effect. All of these cross sections depend on the particle size, shape, phase (i.e., internal structure), and *m*. Comparison of the CRDS-measured cross sections to predictions from an optical model (e.g., Lorenz-Mie theory [LMT] that is applicable to spherical and homogeneous particles) allows the retrieval of both *n* and *k* values at the wavelength of the spectroscopic beam.^26^

Herein, we apply our SP-CRDS technique to determine the real and imaginary components of the complex refractive index, at a wavelength of 405 nm, for aqueous particles containing 4-nitrocatechol. Moreover, a combination of UV/visible spectroscopy measurements of the wavelength-dependent imaginary refractive indices for bulk aqueous solutions of 4-nitrocatechol at different pH, Kramers–Kronig (KK) transformations, and physically based refractive index mixing rules enable the wavelength-dependent values of *n* and *k* to be estimated for the weak acid 4NC and its conjugate base, 4NC^–^.

## 2 Methods

To retrieve the real and imaginary components of the complex refractive index for single, homogeneous aerosol particles containing 4-nitrocatechol, we accurately determine extinction cross sections for evolving particle sizes by SP-CRDS and compare to LMT calculations. Section 2.1 summarizes our SP-CRDS measurement methods, and Section 2.2 describes our UV/visible absorption spectroscopy measurements for bulk aqueous solutions, from which the wavelength-dependent imaginary refractive index is determined.

### 2.1 Single Particle Cavity Ring-Down Spectroscopy

SP-CRDS was used to measure size-dependent extinction cross sections for single, light-absorbing aerosol particles levitated within an LEQ trap. The particle size was retrieved from concurrent elastic light scattering measurements. Thorough descriptions of all the instrument components and experimental approach are provided in our previous publications.^26−28^ Briefly, bulk solutions of 4-nitrocatechol (Sigma-Aldrich, CAS code: 3316-09-04, batch number BCBS2751 V; ≥96%) were prepared with HPLC plus water (Sigma-Aldrich, CAS code: 7732-18-5; pH ∼ 6.7 at 20 °C). The pH values of the solutions were determined to be 5.8–5.9 using a pH meter. The measurements of pH were made within ∼30 min of solution preparation; slow changes in the absorption spectra were observed over time scales of ∼24 h (see Section S2.2 of the Supporting information). The solutions were loaded into a droplet-on-demand dispenser with a 20 μm diameter orifice, from which aerosol droplets with initial diameters 20–25 μm were generated (also within ∼30 min of solution preparation). A small ion imbalance was imparted to the droplets by positioning the droplet dispenser within ∼2–5 mm of an induction ring-electrode to which a 100–300 V voltage was applied, giving the droplets a charge of several femtocoulombs.^28^

The charged aqueous droplets containing 4-nitrocatechol were dispensed into an LEQ trapping cell. The LEQ consisted of four vertically mounted cylindrical rods arranged in a square array, with a ring-electrode located directly below. The droplets fell down the central axis of the LEQ, along the line of stability created by the application of matched AC voltages to diametrically opposite rod pairs. The droplets came to rest as their weight and the drag force, resulting from a downward flow of N_2_ gas used to control the ambient relative humidity (RH), were balanced by the electrostatic repulsion from a DC voltage applied to the ring-electrode. The dispensed droplets were trapped and levitated in the LEQ cell, and they rapidly equilibrated with the ambient RH (>75%; see Section 3.1 for discussion of this lower limit), to give particles with an initial equilibrated radius of *r* ≈ 1.5 μm. The particles then evaporated steadily over time (see Figure S4 of the Supporting information), with the loss of organic solute molecules accompanying the evaporation of water molecules concomitant with the hygroscopicity of the particle at a given RH.

The position of a single levitated particle was manipulated by adjustment of the ring-electrode voltage to control the particle height, and the horizontal position was optimized using a translation stage supporting the LEQ trap, to position the particle precisely at the center of our 405 nm CRDS spectrometer for measurement of the single particle extinction cross sections (see Section S1.1 of the SI). The particle position along the central axis of the LEQ was then monitored using an imaging camera and maintained using a constant feedback loop that adjusted the DC voltage applied to the ring-electrode (e.g., as the weight of the particle changed because of evaporation). The approach to retrieve the real and imaginary components of the complex refractive index from SP-CRDS measurements of particle size-dependent extinction cross sections is described in Section S1.1 of the Supporting information.

Concurrent with the CRDS measurements, the Gaussian beam output from a 632.8 nm wavelength HeNe laser (ThorLabs HNL210LB; 21 mW) propagated vertically upward along the central axis of the LEQ trap, illuminating the levitated particle. A camera coupled to a 20× long working distance objective with a numerical aperture of 0.42 recorded images of the perpendicularly polarized elastically scattered light intensity. Light scattering measurements were recorded over the angular range ∼65.2–114.8° at a frame rate of ∼10 Hz and converted to one-dimensional relative intensity distributions in the perpendicularly polarized scattered light intensity with scattering angle, i.e., phase functions. The particle radius and complex refractive index at 632.8 nm were retrieved by fitting the phase functions to LMT predictions (see ref (29)). The 632.8 nm laser was chosen to avoid particle heating, which could impact the particle properties (see Section S4 of the Supporting information).

### 2.2 UV/Visible Spectroscopy

A concentrated stock solution (1 mM) of 4-nitrocatechol was prepared with HPLC plus water. This concentration is more than an order of magnitude less than the bulk solubility limit for 4-nitrocatechol of 12.2 g L^–1^ (equivalent to 78 mM) reported by Amugoda et al.^30^ The experimental solutions were prepared by diluting aliquots of the stock solution to 50 μM and adjusting the pH with either aqueous HCl or NaOH. A pH meter (Hanna Instruments HI9811-5) measured the temperature-dependent pH values with a resolution in pH of 0.1 and with an accuracy of ±0.1. A GENESYS 10S UV–vis spectrophotometer (Thermo Scientific) was used to measure the absorbance of the solutions over the wavelength range 200–800 nm. For the reasons described in Section 2.1, these measurements were made within ∼3 h of the creation of the stock solution. The ambient temperature for the measurements was 20 ± 1 °C.

## 3 Results and Discussion

Section 3.1 presents CRDS-measured size-dependent extinction cross sections for single light-absorbing aqueous particles containing 4-nitrocatechol and the particle complex refractive indices at a wavelength of 405 nm retrieved from comparison of the measured cross sections to LMT predictions. Additionally, the real refractive indices of the particles that were determined from elastic light scattering measurements at a wavelength of 632.8 nm are provided. Section 3.2 reports the UV/visible absorption spectra for bulk aqueous solutions of 4-nitrocatechol, as a function of the solution pH. A physically based refractive index mixing rule was used to determine the wavelength-dependent imaginary refractive indices for 4NC, 4NC^–^ and 4NC^2–^. Section 3.3. reports the wavelength-dependence of the real refractive indices for 4NC and 4NC^–^, estimated from fitting the corresponding imaginary refractive index spectra to predictions from an analytical function known as the critical point model that is fully KK consistent.^31,32^ These KK inversions were constrained using real refractive index values determined from our SP-CRDS and elastic light scattering measurements.

### 3.1 Aqueous Particles Containing 4-Nitrocatechol

**Figure 1:**
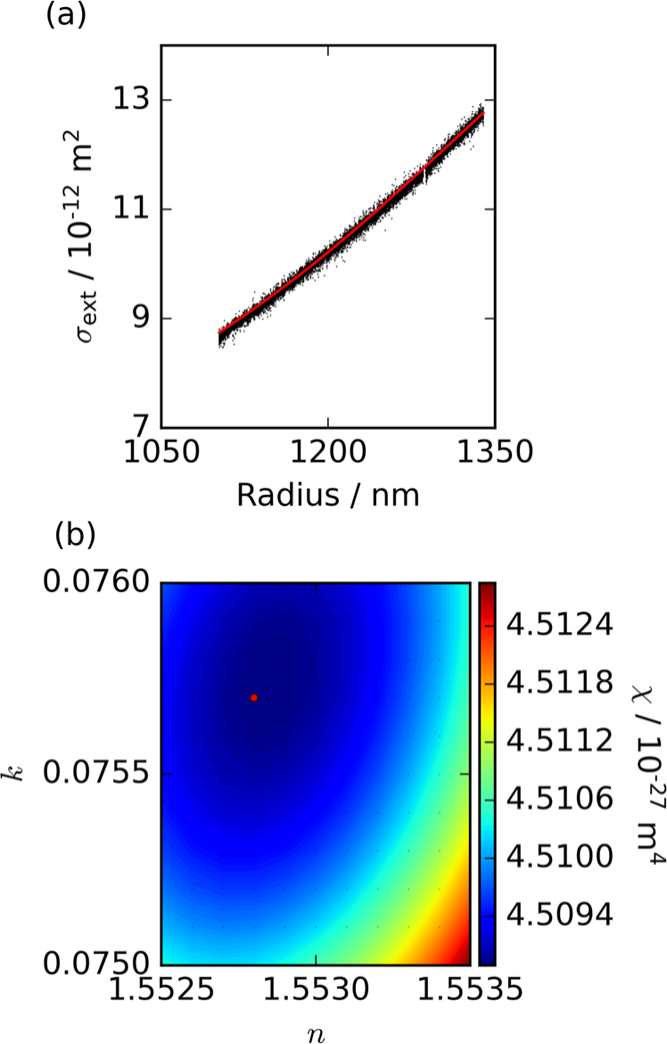
(a) Measured (black points) particle size-dependent variation in extinction cross sections for an aqueous particle containing 4-nitrocatechol held in an 89.5% RH environment. The cross sections have been averaged to a 1 Hz sampling rate and are overlaid with the best-fit LMT prediction (red line). (b) The corresponding contour plot depicting the variation in the merit function with *n* and *k*, for optimized values of beam waist (254.8 μm) and the radius correction factor (1.0019). The red dot indicates the minimum in the retrieved merit functions.

Particle size-dependent extinction cross sections were measured using SP-CRDS for aqueous particles containing 4-nitrocatechol, each levitated in different but constant RH environments. The temporal evolution in particle size was driven by the slow evaporation of 4-nitrocatechol and the subsequent gas-particle partitioning of water to maintain equilibrium between the particle water activity and the ambient RH. Therefore, the composition of each particle was invariant with change in its size. Total measurement times were >5 h for a particle evaporating from a radius of ∼1400 to ∼1000 nm. Figure 1(a) shows the measured particle size-dependent variation in extinction cross-section for an aqueous particle containing 4-nitrocatechol levitated in an 89.5% RH environment. Absorption by this particle dampens the sharp resonance and oscillatory interference contributions to the extinction cross sections.^26,33^ These data represent the most damped size-dependent extinction cross sections reported to date using the SP-CRDS approach, indicative of a large value for *k* at the 405 nm wavelength for these particles. The measured cross sections were fit to LMT predictions according to the description in Section S1.1 of the Supporting information, with the best-fit LMT variation in cross sections shown in Figure 1(a). Figure 1(b) presents the corresponding contour plot depicting the variation in the merit function (χ) with *n* and *k*. The merit function was used to assess the agreement between the measured and predicted cross sections. The other parameters varied in the fits were the beam waist of the cavity mode and a multiplicative correction factor applied to the retrieved particle radius (see Section S1.1 of the Supporting information). The contour plot demonstrates a single, well-defined minimum.

**Figure 2:**
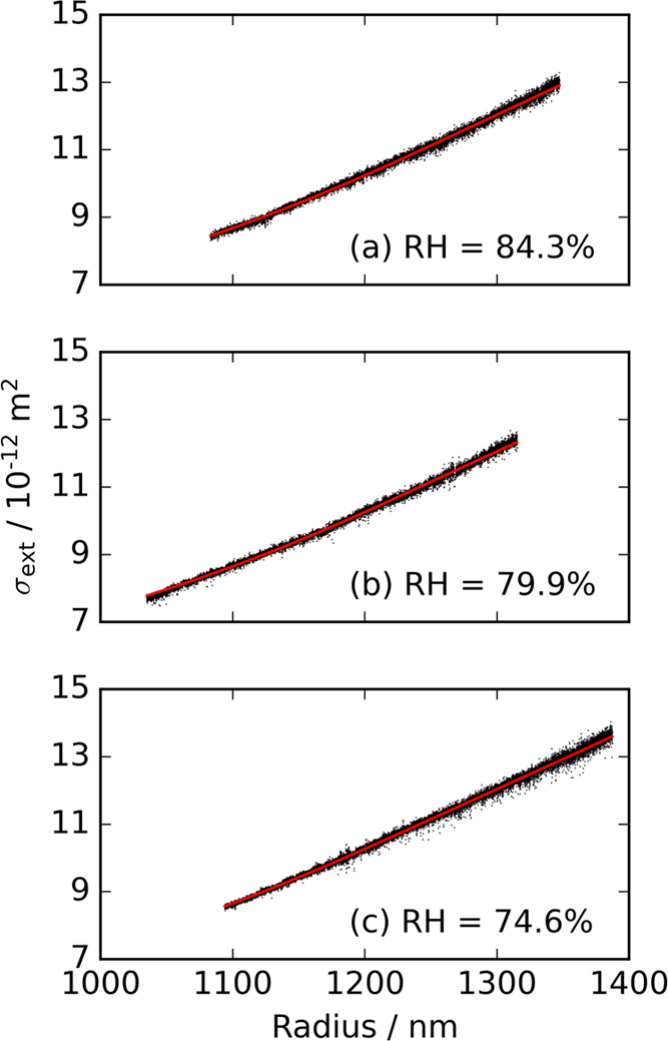
Measured (black points) particle size-dependent variation in extinction cross sections for three different aqueous particles containing 4-nitrocatechol, held in (a) 84.3, (b) 79.9, and (c) 74.6% RH environments. The cross sections have been averaged to a 1 Hz sampling rate and are overlaid with the corresponding best-fit LMT prediction (red line).

Figure 2 shows the CRDS-measured particle size-dependent variation in extinction-cross sections for three further aqueous particles containing 4-nitrocatechol levitated in 84.3, 79.9, and 74.6% RH environments. The corresponding contour plots are provided in Section S1.2 of the Supporting information. The RH at which efflorescence is observed for aqueous particles containing 4-nitrocatechol has been reported to be ∼70%.^25^ To avoid formation of any inhomogeneities within the aerosol particle, the RH surrounding the particles never extended below this value. Section S1.4 of the Supporting information presents the liquid-state vapor pressure for 4-nitrocatechol determined using the measured temporal evolution in particle size for the studied particles. The value reported here (2.33 ± 0.19 × 10^–4^ Pa) is in reasonable agreement with that given elsewhere.^30^

**Figure 3:**
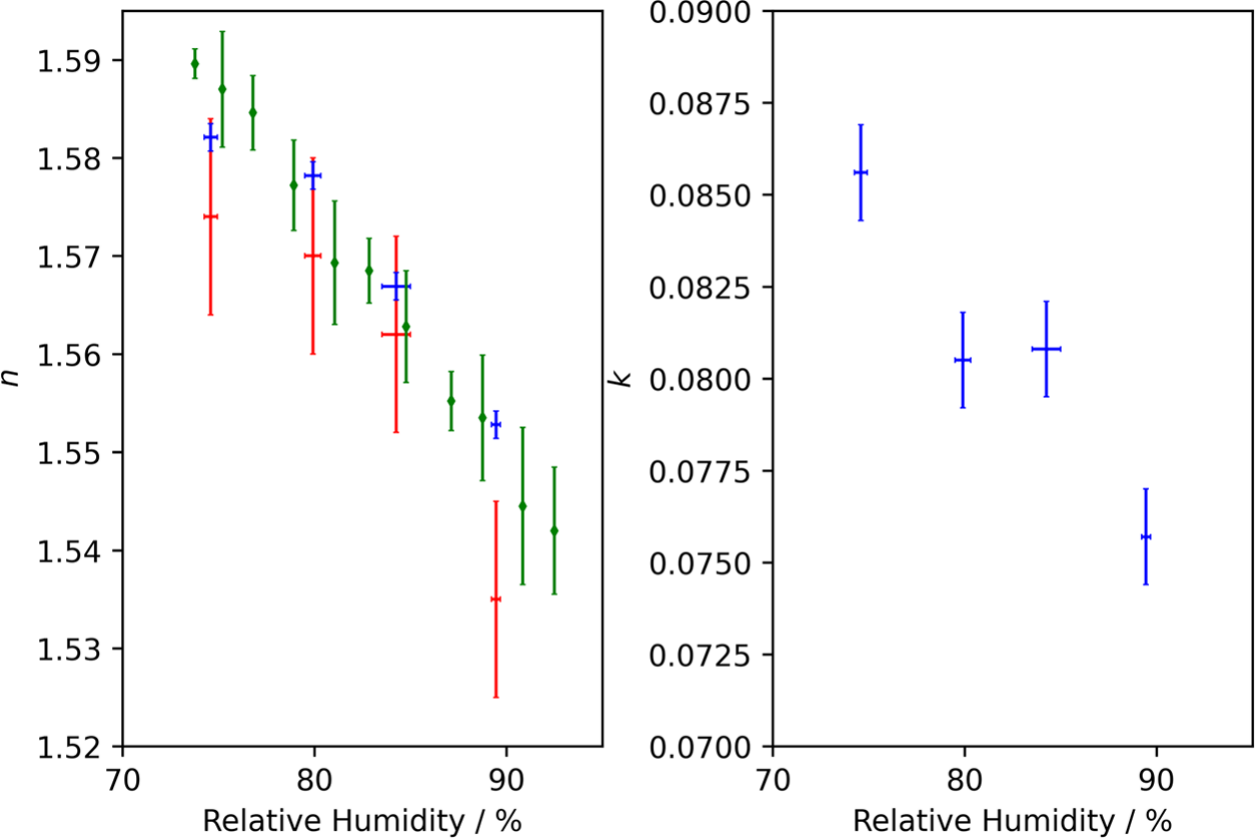
Real (left) and imaginary (right) components of the complex refractive index at a wavelength of 405 nm, retrieved from CRDS-measured particle size-dependent variations in extinction cross sections, for four 4-nitrocatechol-containing aqueous particles levitated in different RH environments (blue points). The corresponding real refractive indices at a wavelength of 632.8 nm, determined from angularly resolved elastic light scattering measurements, are shown by red points. The real refractive index components of aqueous particles containing 4-nitrocatechol determined by Price et al. at a wavelength of 589 nm are shown by green diamonds.^25^

Figure 3 shows the retrieved real and imaginary components of the complex refractive index for the four different particles. At higher RH values, the particles contain more water to maintain equilibrium with the surrounding gas. Water has a pure component real refractive index of 1.3388 and an imaginary refractive index of ∼10^–9^ at a wavelength of 405 nm and temperature of 298.15 K.^34^ These refractive index components are lower than values for typical organic and inorganic solutes.^35−39^ Therefore, the values of *n* and *k* for the particles are expected to decrease with an increase in RH. The error bars for the RH represent the standard deviations in the RH values that were recorded continuously at a sampling rate of 1 Hz during each experiment. Also depicted are the real refractive index values determined at a wavelength of 632.8 nm from concurrent elastic light scattering measurements. Discussion of the error bars in the retrieved real and imaginary components of the complex refractive index is provided in Section S1.3 of the Supporting information.

Figure 3(a) compares our real refractive index values for aqueous particles containing 4-nitrocatechol with those determined by Price et al. at a wavelength of 589 nm from broadband light scattering measurements. The good agreement indicates that the compositions of the aqueous particles are the same in the two studies. This is expected as the composition should depend only on the chemical identity of the solute and the ambient relative humidity. Systematic discrepancies between these RH-dependent refractive index data should arise only from the different choices of optical wavelength and errors in the measurement of the RH at the center of the LEQ trapping cells.

The ratio of the protonated, singly and doubly deprotonated forms of 4-nitrocatechol present within the aerosol particles depends on the pH of the particles and therefore on the activity of H^+^. The activity coefficient of H^+^ is expected to deviate from unity because nonideal interactions can be particularly significant for aerosol particles, for which supersaturated solute concentrations are possible, and may exceed concentrations in bulk solutions. Indeed, the bulk solubility limit for 4-nitrocatechol in water is ∼1–2% by mass while the particles studied here are composed of up to ∼75% 4-nitrocatechol by mass (see Section 3.2). Moreover, the direct measurement of aerosol particle pH is challenging. One simple approach involves the impaction of aerosol particles onto pH-indicator paper,^40^ but requires total particulate masses of milligrams while the single particles studied in our work have masses ∼10^–12^–10^–11^ g. Another direct approach is to use Raman spectroscopy to determine the concentrations of acid and conjugate base species contained within a particle.^41,42^ For the studies reported here, no applicable approach for direct pH determination in the aqueous particles containing 4-nitrocatechol was available. Further discussion of the particle acidity and composition is provided in later sections.

### 3.2 Wavelength-Dependent Imaginary Refractive Index for 4NC, 4NC^–^, and 4NC^2–^

**Figure 4:**
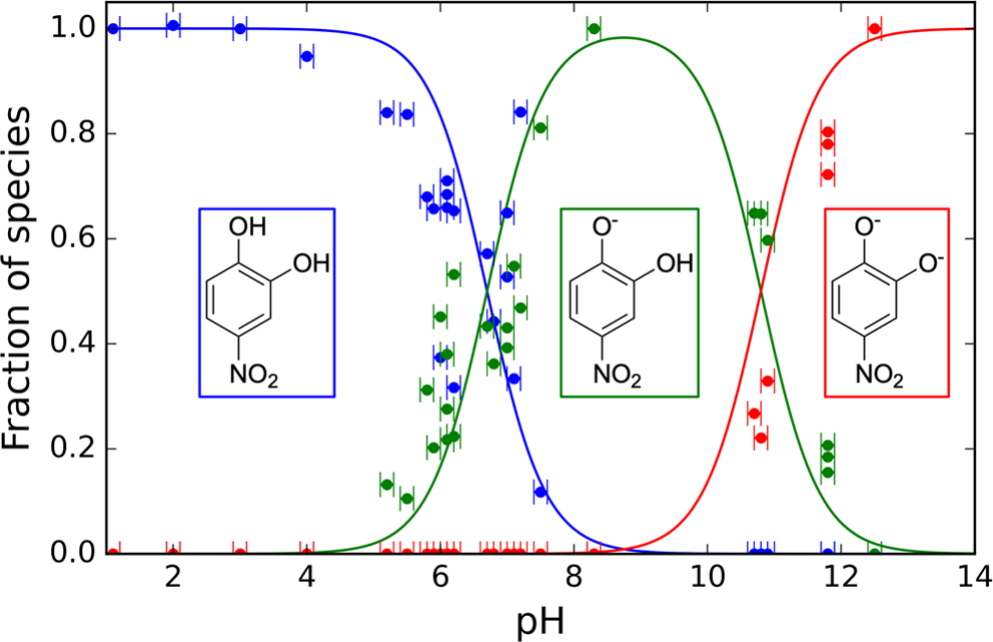
pH dependent fractions of 4NC, 4NC^–^, and 4NC^2–^ in aqueous solution are depicted in blue, green and red, respectively. The points represent measured fractions determined using UV/visible spectroscopy (see Section S3.1 of the Supporting information for details), while the lines represent predictions from a Henderson–Hasselbalch model incorporating reported p*K*_a_ values.^23^ The error bars represent the accuracy of the pH meter used to measure solution pH (see Section 2.2).

The optical properties of 4-nitrocatechol depend on the pH of an aqueous particle, and therefore we must first establish an approach to determine the fractional ratios of the 4NC, 4NC^–^ and 4NC^2–^ species at a given pH. Figure 4 shows the fractional composition of the solute (i.e., the fractions of 4NC, 4NC^–^ and 4NC^2–^) present in bulk aqueous solutions of 4-nitrocatechol at different pH values. The expressions for calculating the fractions of acid–base species within the solutions are derived from the acid dissociation constant (see Section S2.4 of the Supporting information) and use the p*K*_a_ values reported in Scheme 1.

Section S2.1 of the Supporting information presents the UV/visible absorption spectra for aqueous solutions of 4-nitrocatechol, obtained for a broad range of pHs. The spectra show an increase in absorbance at longer (visible) wavelengths with increasing pH because of the greater fractions of 4NC^–^ and 4NC^2–^. The data points in Figure 4 represent the fractions of each species in aqueous solution determined by applying a spectral decomposition algorithm to our UV/visible spectra (see Section S3.1 of the Supporting information). This spectral decomposition used the pure component spectra for 4NC, 4NC^–^, and 4NC^2–^ discussed later in this section. The measured points validate the Henderson–Hasselbalch model, which is used hereafter to determine the fractions of 4NC, 4NC^–^, and 4NC^2–^. The measured absorbance *A* is related to the absorption coefficient (α_abs_) through the Beer–Lambert law, with the imaginary refractive index for the aqueous solution given by the equation
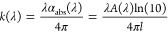
1in which λ is the wavelength of light and *l* is the path length of the UV/visible probe beam through the sample (1 cm).^33^ Application of a physically based refractive index mixing rule (a linear weighting of the imaginary refractive indices of mixed components by their mass fractions) allowed the wavelength-dependent imaginary refractive indices for the pure component 4NC, 4NC^–^, and 4NC^2–^ species to be deduced.^27^ This mass fraction mixing rule for the aqueous solutions of 4-nitrocatechol can be written as
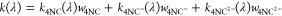
2with *k* being the imaginary refractive index of the particle, and *k*_4NC_, *k*_4NC^–^_, *k*_4NC^2–^_ the imaginary refractive indices of 4NC, 4NC^–^, and 4NC^2–^, respectively. The *w*_4NC_, *w*_4NC^–^_, *w*_4NC^2–^_ are the corresponding mass fractions, with the sum of these three mass fractions and the mass fractions of water and either HCl or NaOH equal to one. The values of these mass fractions are dependent on the pH of the studied solutions, and the pH dependence of the overall absorption spectra allows the spectral components to be separated. The imaginary refractive index of water is <10^–7^ across the 200–800 nm wavelength range of our UV/visible spectra so is omitted from eq 2. Similarly, the terms pertaining to HCl and NaOH, that were used to adjust the solution pH, are omitted because their imaginary refractive indices are negligible across the visible spectrum. The combined mass fraction for 4NC, 4NC^–^, and 4NC^2–^ was calculated from the known total solute concentration in our prepared samples (50 μM of 4-nitrocatechol and the concentration of either HCl or NaOH required to attain the desired solution pH). Then, the mass fractions of 4NC, 4NC^–^, and 4NC^2–^ were determined using the Henderson–Hasselbalch model to predict fractional composition of aqueous solutions of 4-nitrocatechol as a function of pH (see modeled curves in Figure 4) and applying those fractions to the calculated combined mass fraction for the three species. The wavelength-dependent imaginary refractive indices for the pure 4NC, 4NC^–^, and 4NC^2–^ components were calculated from the UV/visible absorption spectrum for aqueous solutions of 4-nitrocatechol and either HCl or NaOH with pH = 1.1, 8.3, and 12.5, respectively (see Figure S4 of the Supporting information). These calculations are discussed in Section S2.3 of the Supporting information.

**Figure 5:**
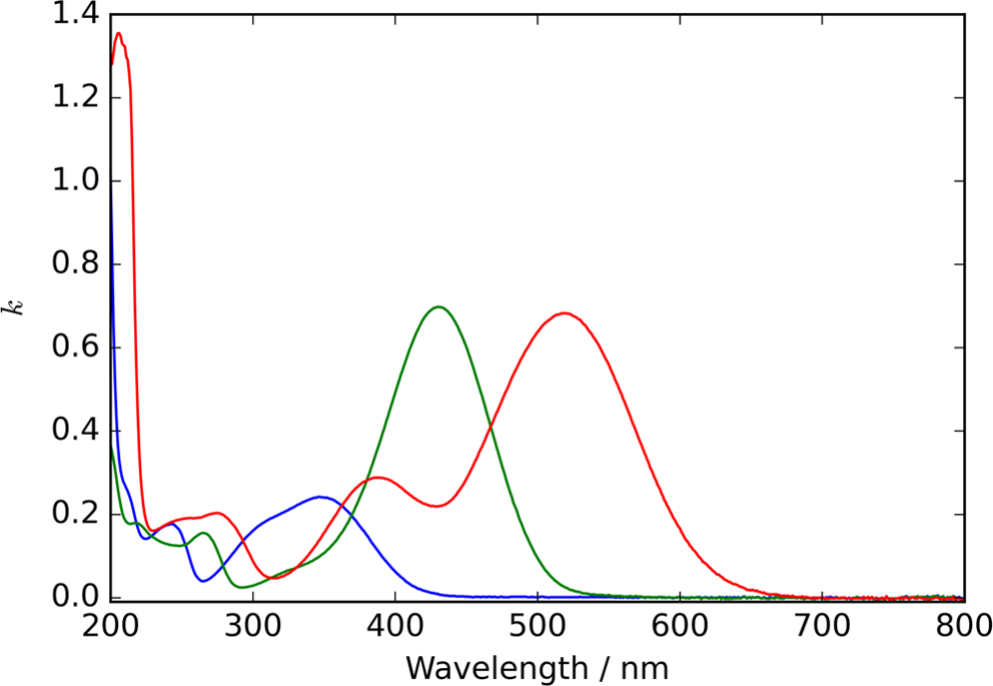
Wavelength-dependent imaginary refractive indices for 4NC (blue line), 4NC^–^ (green line), and 4NC^2–^ (red line), deduced from decomposition of UV/visible absorption spectra for bulk aqueous solutions of 4-nitrocatechol at different pH values.

Figure 5 depicts the wavelength-dependent imaginary refractive indices for the pure 4NC, 4NC^–^, and 4NC^2–^ species obtained from this analysis. The accuracy of the pH meter used (±0.1) was not accounted for as the resultant uncertainty in the imaginary refractive indices had a negligible impact on assessments of particle composition described later. The imaginary refractive indices at a wavelength of 405 nm measured using UV/visible spectroscopy were 0.047 for 4NC and 0.545 for 4NC^–^. These values indicate that the single aqueous particles studied using CRDS (Section 3.1), for which we determined *k* values >0.075 (see Figure 3), contained some 4NC^–^. The particles were assumed to contain water, 4NC, and 4NC^–^ only, with no appreciable fraction of 4NC^2–^ (see Section S4 of the Supporting information for justification).

The mass fraction mixing rule can be applied to predict the fractions of 4NC and 4NC^–^ within the single particles probed in Section 3.1. The mass fractions of water, 4NC, and 4NC^–^ were calculated using the radial growth factors for aqueous particles containing 4-nitrocatechol reported by Price et al.^25^ A value for the density of 4NC has not been reported. Therefore, the density of 4NC was assumed to be the same as a similar nitroaromatic compound, 4-nitrophenol (1.48 g cm^–3^).^43,44^ The densities of 4NC and 4NC^–^ were taken to be the same. Then, using the values of *k*_4NC_ and *k*_4NC^–^_ measured by UV/visible spectroscopy, and varying the values of *w*_4NC_ and *w*_4NC^–^_ (to ratios corresponding to the speciation curves shown in Figure 4) until the mass fraction mixing prediction of *k* matched the measured values for our single aqueous droplets, the solute fractions of 4NC and 4NC^–^ within the particles were estimated to be 0.870 ± 0.004 and 0.130 ± 0.004, respectively. The predicted fractional composition of solute did not change appreciably between the four aqueous 4-nitrocatechol containing particles studied (i.e., over the range of total solute concentrations for the particles levitated in different RH environments); the fractional composition of solute for each of the studied particles was within the uncertainty of the measurements. This uncertainty comes from the accuracy of the CRDS-determined imaginary refractive indices for the particles (±0.0013; see Section S1.3 of the Supporting information). Contribution to this uncertainty from the assumed density of 4NC was omitted. Moreover, uncertainties in the radial growth factors reported by Price et al. were not propagated through to our final values because they had a negligible effect on the estimated fractions of 4NC and 4NC^–^ (<0.1%).^25^ According to Figure 4, the fractions of these two species indicate a particle pH of 5.87 ± 0.02 (noting that the calculations for Figure 4 neglect nonideal behavior of H^+^; i.e., that the activity coefficient of H^+^ is unity).

### 3.3 Wavelength-Dependent Real Refractive Indices for 4NC and 4NC^–^

#### 3.3.1 Critical Point Model

The KK relations can be used to relate wavelength-dependent distributions for the real refractive index to the imaginary refractive index, and vice versa.^33^ Here, a parametrized and fitted analytical function that is KK consistent allows the real component of the complex refractive indices for 4NC and 4NC^–^ to be estimated from measured imaginary refractive index distributions shown in Figure 5. One common approach is to represent the optical response of a medium with a set of oscillator functions. The wavelength-dependent imaginary refractive index is often modeled as a sum of Lorentzian oscillators.^25,45,46^ However, these models often describe poorly the imaginary refractive index because the Lorentzian line shape decays slowly, resulting in persistent tails. The introduction of additional oscillators can negate this effect, but these additional oscillators are inherently unphysical.

The critical point model has been used to model successfully the dielectric functions of semiconductors,^32^ and molecular polarizability of dye molecules, and shown to be KK consistent.^31^ The approach taken here is to use a critical point model that describes the optical transitions of a species as the sum of a set of critical point line shapes.^47^ The real and imaginary components of the complex refractive index are given by evaluating the real or imaginary parts of the equation:
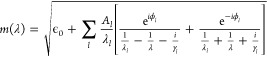
3in which *A*_*l*_ is the amplitude of the contribution from oscillator *l*, λ_*l*_ is the resonance wavelength, and γ_*l*_ is a broadening parameter. The term ϕ_*l*_ denotes a phase factor that allows an asymmetric line shape, giving greater control over the decay of the peak than for the models using Lorentzian oscillators. Finally, the parameter ϵ_0_ is the short-wavelength limit (λ → 0) of the dielectric constant, which accounts for contributions to the dielectric function originating from optical transitions at shorter wavelengths than the spectral range studied here using UV/visible spectroscopy (i.e., at λ < 200 nm).

The wavelength-dependent imaginary refractive indices for 4NC and 4NC^–^ shown in Figure 5 were fitted to the critical point model function using a differential evolution (DE) algorithm.^48^ For each species, the algorithm optimized the value of the constant ϵ_0_ and the values *A*_*l*_, λ_*l*_, γ_*l*_, and ϕ_*l*_ describing each oscillator. For 4NC, the best-fit was obtained by incorporating four critical point oscillators (i.e., up to *l* = 4), and the values for the oscillator parameters and ϵ_0_ were found by minimizing the sum of squared residuals between the modeled and measured wavelength-dependent imaginary refractive indices. The 4NC^–^ analysis used the same methodology but required six critical point line shapes (i.e., up to *l* = 6) to model the absorption spectrum. The values of the parameters describing these best-fit critical point models are provided in Section S3.3 of the Supporting information.

#### 3.3.2 Wavelength-Dependent Real Refractive Indices Constrained by SP-CRDS Measurements

The KK inversions using the critical point model can be made more robust if constrained by pure-component (4NC and 4NC^–^) *n* values determined from analysis of the SP-CRDS measurements for aqueous particles containing 4-nitrocatechol shown in Figure 3. This section describes the deduction of the pure component values, then uses these to constrain the best-fit values for the parameters ϵ_0_, *A*_*l*_, λ_*l*_, γ_*l*_, and ϕ_*l*_ during the application of the DE algorithm to fit the measured imaginary refractive index spectra.

The real refractive indices of 4NC and 4NC^–^ are expected to be comparable at wavelengths much larger than their visible-wavelength absorption bands because of the similarity between the two molecules. Therefore, the differences between the wavelength-dependent real refractive index values of 4NC and 4NC^–^ were first estimated by setting their values to be equal at a wavelength of 1 mm, arbitrarily assigned a value of unity. Increasing the value of this wavelength further had a negligible impact on the differences between the real refractive index values of 4NC and 4NC^–^ predicted below. The critical point oscillator model was used to reveal the wavelength distributions for their real refractive indices across the visible spectrum. The real refractive index of 4NC was estimated to be 0.482 smaller than that for 4NC^–^ at a wavelength of 405 nm from this assessment (see Section S3.2 of the Supporting information). Knowledge of this difference between the real refractive indices of the two species allows the estimation of their values from the analysis that follows.

The real refractive indices of the two pure component species 4NC and 4NC^–^ were estimated from the 405 nm SP-CRDS determined particle refractive indices (Figure 3) using the mole fraction (*x*) weighting of molar refraction mixing rule
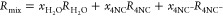
4in which *R*_mix_, *R*_H_2_O_, *R*_4NC_, and *R*_4NC^–^_ are the molar refractions of the internally mixed particle, water, 4NC, and 4NC^–^, respectively, and the *x* values are the corresponding mole fractions. The molar refraction is defined by
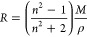
5in which *M* is the molecular weight and ρ is the mass density.^24^ For physically realistic values of the refractive index, it has been demonstrated that eq 5 is KK consistent.^49^ The pure component molar refraction for water was calculated from the known *n* at a wavelength of 405 nm, and values of *M* and ρ for water; 1.3388,^34^ 18.0 g mol^–1^, and 0.997 g cm^–3^, respectively. To determine *n*_mix_ from *R*_mix_, the mean molecular weight (*M*_mix_) and mass density (ρ_e_) for the internally mixed particles were predicted using
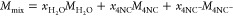
6and
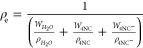
7in which ideal mixing is assumed and *w*_H_2_O_, *w*_4NC_, and *w*_4NC^–^_ are the mass fractions of water, 4NC and 4NC^–^, respectively. The mole and mass fractions of each of the species were calculated using the radial growth factors for aqueous particles containing 4-nitrocatechol reported by Price et al., and the fractions of 4NC and 4NC^–^ determined in Section 3.2.^25^ The molecular weights of 4NC and 4NC^–^ used were 155.11 and 154.11 g mol^–1^, respectively. The density of 4NC and 4NC^–^ were the same as those provided in Section 3.2. The 405 nm real refractive indices for 4NC and 4NC^–^ were then calculated, while maintaining the estimated difference between the refractive indices of the two species (0.482). The mean values of the real components of the complex refractive indices for 4NC and 4NC^–^ obtained from all four particles at a wavelength of 405 nm were 1.797 ± 0.017 and 1.313 ± 0.017 respectively. The uncertainty represents the accuracy of the measurements estimated from propagation of uncertainties in the particle real refractive index determined from SP-CRDS measurements, the fractions of 4NC and 4NC^–^ estimated in Section 3.2, and the radial growth factors reported by Price et al. (∼0.01); this uncertainty analysis is described in Section S3.3 of the Supporting information.^25^

**Figure 6:**
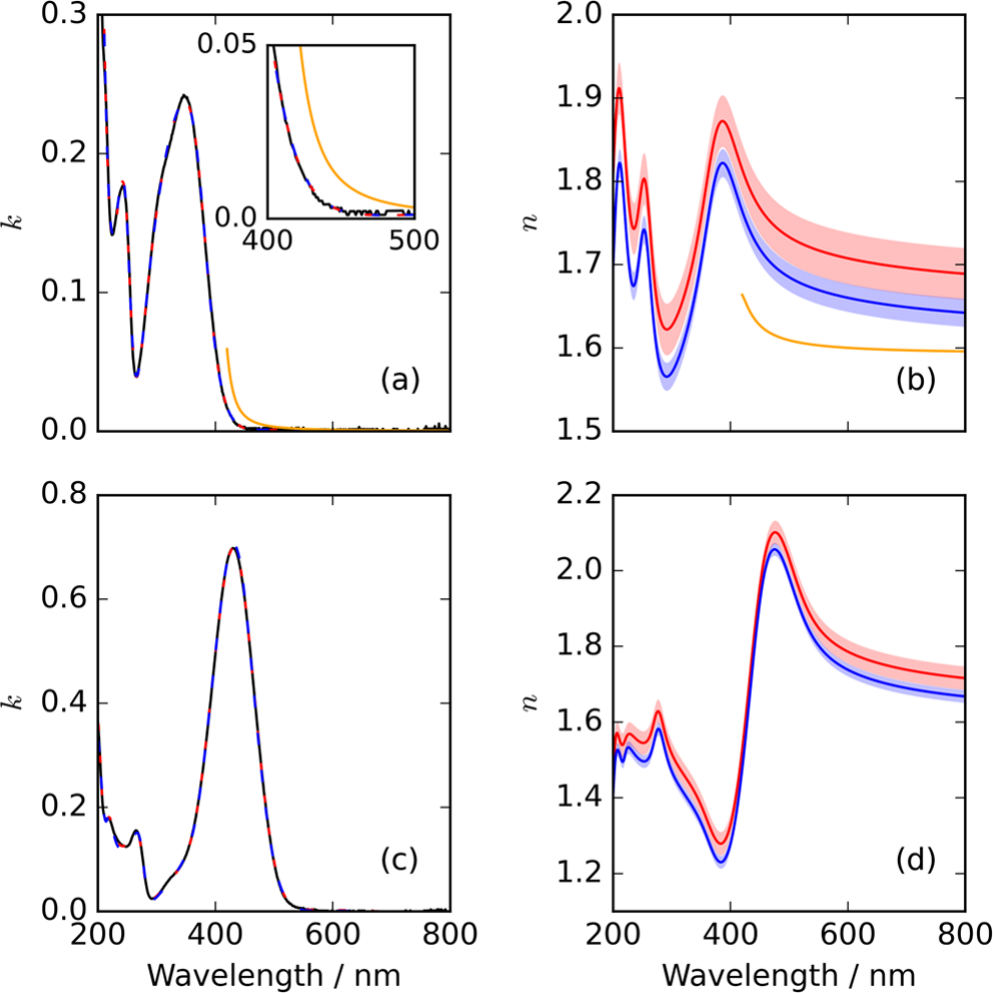
Kramers–Kronig analysis of the imaginary and real refractive indices of pure component 4-nitrocatechol species. (a) The measured wavelength-dependent imaginary refractive index for 4NC (black line) is overlaid with the best-fit obtained using the critical point model. The resulting predicted real refractive index distributions are given in panel (b); the inversions were constrained using experimentally determined real refractive index values at either 405 nm (blue dashed/solid lines; see Section 3.3.2) or 632.8 nm (red dashed/solid lines; see Section 3.3.3). The corresponding analysis for 4NC^–^ is depicted in (c, d). The shaded envelopes represent the uncertainties in the determined refractive indices. The orange lines in panels (a, b) show estimates for *n* and *k* taken from Price et al. (see Section 3.3.4).^25^

Figure 6(a) shows the measured wavelength-dependent imaginary refractive index for pure 4NC (i.e., the blue line in Figure 5), and the best-fit critical point prediction (blue dashed line). Figure 6(b) depicts the corresponding wavelength-dependence of the real refractive index predicted from the KK relationship (eq 3) using the critical point model. Additionally, Figure 6(c,d) show the corresponding wavelength-dependencies of the imaginary and real components of the refractive index for 4NC^–^, respectively. The KK inversions were constrained using the pure component real refractive index values at 405 nm estimated above (i.e., 1.797 for 4NC and 1.313 for 4NC^–^). The uncertainties in modeled wavelength-dependent real refractive index values for 4NC and 4NC^–^ are equal to those in the SP-CRDS determined real refractive index values that constrained the inversions.

#### 3.3.3 Wavelength-Dependent Real Refractive Indices Constrained by Elastic Light Scattering Measurements

We repeat the analysis described in Section 3.3.2 using the real refractive index retrievals from the 632.8 nm wavelength elastic light scattering measurements instead of 405 nm SP-CRDS retrievals to constrain the KK inversions. The difference between the real refractive index values of the two species at 632.8 nm was first estimated following the same methodology as in the previous section, with the real refractive index of 4NC predicted to be 0.061 smaller than that of 4NC^–^. The 632.8 nm real refractive indices for 4NC and 4NC^–^ were then calculated using eq 4, while maintaining this difference between the refractive indices of the two species. All of the parameters required to solve for the real refractive indices of the two species were the same as those used in the preceding section, excluding the real refractive index of water at 632.8 nm which was assigned a value of 1.3315.^34^

The mean values of the real components of the complex refractive indices for pure component 4NC and 4NC^–^ obtained from all four aqueous particles at a wavelength of 632.8 nm were 1.703 ± 0.031 and 1.765 ± 0.031, respectively. The uncertainties represent accuracies propagated from the uncertainties in the particle real refractive indices determined from elastic light scattering measurements (see Section S3.3 of the Supporting information), the fractions of 4NC and 4NC^–^ estimated in Figure 4, and the radial growth factors reported by Price et al.^25^ These estimated pure component real refractive indices for 4NC and 4NC^–^ were used to constrain the critical point inversion procedure for determining the wavelength-dependent real refractive indices of the two species. The resulting wavelength-dependent imaginary and real refractive index components of 4NC are compared with the 405 nm SP-CRDS constrained analysis outcomes in Figure 6(a,b). Figure 6(c,d) show the corresponding outcomes for 4NC^–^. The uncertainties in modeled wavelength-dependent real refractive index values for 4NC and 4NC^–^ are equal to the uncertainty in the experimentally determined (at 632.8 nm) real refractive index values that constrained the inversions.

#### 3.3.4 Examination of Wavelength-Dependent Complex Refractive Indices for 4NC and 4NC^–^

The KK-predicted wavelength-dependent real refractive indices for 4NC, using two different experimentally determined real refractive index values as constraints, showed slight differences (see Figure 6(b)), as did the corresponding predictions for 4NC^–^ (Figure 6(d)). These differences could be caused by an underestimate in the uncertainty assigned to the real refractive indices retrieved from elastic light scattering measurements at 632.8 nm (see Section S1.5 of the Supporting information). Moreover, the assumption that the compounds within the studied particles were ideally mixed may contribute to the discrepancy; the estimated solute mass fraction within the particles was over an order of magnitude greater than the bulk solubility limit. Therefore, it is likely that there were nonideal interactions between solute molecules. Cai et al. showed that the deviation between experimentally determined densities of binary aqueous–organic mixtures and density predictions using the ideal mixing rule can reach values of ±4%.^50^

Price et al. used Mie resonance spectroscopy to analyze broadband light scattering spectra from aqueous particles containing 4-nitrocatechol that were levitated in an LEQ trap.^25^ The real and imaginary components of the complex refractive index for pure 4NC were determined using a single Lorentzian oscillator to describe the tail of the strong absorption bands in the near-UV. The best-fit solutions for the wavelength-dependent real and imaginary components of the complex refractive index for pure 4NC are shown by the orange lines in Figure 6(a,b). These estimations from Price et al. follow the same trends as those predicted by our analysis. However, the real refractive index values obtained by Price et al. are ∼0.1 lower than the values estimated here across the studied spectral range. Additionally, the imaginary refractive indices reach values that are up to ∼0.04 greater than those predicted here. Importantly, the values for the pH of the aerosol particles studied by Price et al. were not specified, and it was assumed the particles were composed of 4NC and water only. The implicit assumption that 4NC^–^ was not present within their particles may contribute to the differences with the analysis outcomes reported here. However, their reported light-induced heating of aqueous 4-nitrocatechol-containing particles on illumination with an unfocused 532 nm laser beam points to the presence of 4NC^–^ because 4NC does not absorb at 532 nm whereas 4NC^–^ does (see Figure 5). The accord between the RH-dependent real refractive indices shown in Figure 3(a) indicates that the compositions of their particles were the same as those interrogated in our work, as expected from equilibrium thermodynamic considerations.

Price et al. determined a real refractive index for pure 4NC of 1.7 at a wavelength of 589 nm, derived from application of a volume fraction mixing rule to the real refractive index values obtained for aqueous particles containing 4-nitrocatechol using broadband light scattering.^25^ This analysis also assumed a negligible fraction of 4NC^–^ in their studied aerosol particles. Despite these differences in the analysis, the value of the real refractive index determined by Price et al. is in good agreement with our KK predicted real refractive index at 589 nm (see Figure 6(b)).

## 4 Conclusions

We have reported the wavelength-dependent real and imaginary components of the complex refractive index for 4NC and 4NC^–^, which are the two forms of 4-nitrocatechol relevant to atmospheric aerosols. In the visible region, 4NC^–^ is a stronger absorber than 4NC, and its absorption bands are shifted to longer wavelengths. The parameters describing absorption of visible light by 4NC^–^ reported here, with *k* reaching values comparable to those reported for black carbon (∼0.8), suggest that the warming effects of light-absorbing organic carbon (i.e., BrC) species may be underpredicted if the pH dependence of the absorption spectra is not considered. Moreover, a significant fraction of the 4-nitrocatechol constituents of cloud droplets is likely to exist as 4NC^–^ because the pH of cloud droplets can be above the p*K*_a_ of 4NC, which may lead to a semidirect effect that reduces cloud cover in the mid-to-lower troposphere, amplifying the atmospheric warming effect of nitroaromatic compounds. The wavelength-dependent real and imaginary components of the complex refractive indices for 4NC and 4NC^–^ reported here will improve the representation of aerosol-light interactions for BrC aerosol in climate models. Further studies are required to explore the wavelength-dependent light absorption for other abundant nitroaromatic compounds.

## Supporting Information Available

Descriptions of our SP-CRDS and UV/visible spectroscopy experiments, and the critical point model; experiments for the qualitative determination of particle composition, and calibration of the capacitance probe used to measure the ambient RH during our SP-CRDS studies (PDF)
