# Supplementary material for: Wavelength- and pH-Dependent Optical Properties of Aqueous Aerosol Particles Containing 4-Nitrocatechol
Source: ACS Earth Space Chem. 2024 Nov 12;8(11):2198–208. doi: 10.1021/acsearthspacechem.4c00179 (PMC11587064; doi:10.1021/acsearthspacechem.4c00179)
Supplement: Supplementary file 1 — sp4c00179_si_001.pdf [file sp4c00179_si_001.pdf]

Supporting Information

The wavelength- and pH-dependent optical properties of aqueous aerosol particles containing 4-nitrocatechol

Jamie W. Knight,[1] Josephine E. M. Forsythe,[2] Xu Zhang,[1] Aidan Rafferty,[2] Andrew Orr-Ewing,[1*] Michael I. Cotterell[2*]

[1]School of Chemistry, University of Bristol, Bristol, BS8 1TS, United Kingdom

[2]Department of Chemistry, University of Oxford, Oxford, OX1 3QZ, United Kingdom

*Correspondence to: Michael I. Cotterell (michael.cotterell@chem.ox.ac.uk), Andrew J. Orr-Ewing (a.orr-ewing@bristol.ac.uk)

Table of Contents

S1 Single Particle Cavity Ring-Down Spectroscopy S2

S1.1 Particle Complex Refractive Index Retrieval Algorithm S2

S1.2 Particle Complex Refractive Index Retrievals using SP-CRDS S4

S1.3 Accuracy and Precision in Complex Refractive Index Retrievals using SP-CRDS S5

S1.4 The Vapor Pressure of 4-Nitrocatechol S10

S1.5 Uncertainty in Real Refractive Index Retrievals using Elastic Light Scattering S12

S2 UV/visible Spectroscopy S12

S2.1 Absorption Spectra for Aqueous Solutions of 4-Nitrocatechol S13

S2.2 Temporal Variation in Absorption Spectra for Aqueous Solutions of 4-Nitrocatechol S14

S2.3 Wavelength-Dependent Imaginary Refractive Indices for 4NC, $4NC^{-}$ and $4NC^{2-}$ S15

S2.4 Fractional Composition of 4-Nitrocatechol Species Determined using a Henderson–Hasselbalch model S16

S3 Critical Point Model S17

S3.1 Fractional Composition of 4-Nitrocatechol Species Determined Using a Spectral Decomposition Algorithm S18

S3.2 Differences Between the Wavelength-Dependent Real Refractive Indices for 4NC and $4NC^{-}$ S19

S3.3 Critical Point Lineshapes Describing the Wavelength-Dependent Refractive Index for Pure Component 4NC and $4NC^{-}$ S20

S3.4 Uncertainty in the Real Refractive Indices for 4NC and $4NC^{-}$ Estimated from SP-CRDS and Elastic Light Scattering Measurements S23

S4 The Influence of Illumination Beam Wavelength on the Temporal Evolution in Particle Size S24

S5 Calibration of the Capacitance Probe used for Measurements of Relative Humidity S26

S1 Single Particle Cavity Ring-Down Spectroscopy

Section S1.1 describes our approach to retrieve the real and imaginary components of the complex refractive index of aerosol particles by fitting particle size-dependent extinction cross-sections measured by single particle cavity ring-down spectroscopy (SP-CRDS) to a light scattering model. The contour plots provided in Sect. S1.2 describe the agreement between the measured and modelled cross-sections and show the retrieved complex refractive indices determined for the aqueous particles containing 4-nitrocatechol studied here.

The theoretical accuracy and precision in the real and imaginary components of the complex refractive index retrieved from our SP-CRDS experiments for aqueous particles containing 4-nitrocatechol are determined in Sect. 1.3. These theoretical accuracy assessments model the temporal evolution of particle size for the particles, which requires the vapor pressure of 4-nitrocatechol to be known. Section S1.4 presents the temporal evolution in particle size for each of the four aqueous particles containing 4-nitrocatechol studied and describes how the vapor pressure of 4-nitrocatechol is determined from our experimental measurements. The accuracy of the real refractive indices determined using our elastic light scattering measurements is discussed in Sect. S1.5.

S1.1 Particle Complex Refractive Index Retrieval Algorithm

The method used here to retrieve the real and imaginary components of the complex refractive index of aerosol particles from our SP-CRDS measurements of particle size-dependent extinction cross-sections is identical to that described in Ref. [1] and the implementation of, and motivations for, the approach have been described in detail in Ref. [2]. Briefly, the CRDS-measured ring-down times were binned to 1 s intervals to average out the effects of particle position within the cavity standing wave. The mean ring-down time within those bins was calculated and converted to an extinction cross-section. Extinction cross-section values (σ_{ext}) were deduced from the difference between the measured ring-down time when a particle was present (τ) and for an empty cavity (τ_0) using:[3, 4]

$$\sigma_{\text{ext}} = \left(\frac{1}{\tau} - \frac{1}{\tau_0}\right)\frac{L\pi w_0^2}{2c} \qquad \text{(S1)}$$

in which L is the cavity length (the distance between the two mirrors; 0.8 m), w_0 is the beam waist of the TEM_{00} Gaussian cavity mode at the location of the trapped particle, and c is the

speed of light. A least-squares fit of the CRDS-measured extinction cross-sections (averaged to 1 s intervals) to Lorenz-Mie theory (LMT) predictions was performed and the fits were evaluated using the merit function (χ):

$$\chi = \frac{1}{N}\sum_{i=1}^{N}\left(\sigma_{\mathrm{exp,i}} - \sigma_{\mathrm{Mie,i}}\right)^{2} \tag{S2}$$

in which N was the total number of data points, $\sigma_{\mathrm{exp,i}}$ the measured extinction cross-section at a given particle radius, and $\sigma_{\mathrm{Mie,i}}$ the LMT predicted value for a given complex refractive index at the same particle radius. The fitting parameters describing the complex refractive index input to LMT calculations of extinction cross-section were varied using a grid search algorithm. The real and imaginary components of the complex refractive index were assumed to be constant throughout the experiments, i.e., the compositions of the studied particles were invariant with change in particle size (see Sect. 3.1 of the main manuscript). For the measurements reported here, the range of n and k values initially spanned a large range before sequential reduction to allow retrievals to the nearest 0.0001.

Uncertainties in the length of the cavity, curvature of the CRDS mirrors, and the longitudinal position of the particle within the cavity TEM_{00} mode cause uncertainty in the value of the beam waist (w_0) of the intracavity beam. Therefore, the value of the beam waist used to convert the averaged ring-down times to cross-sections (Eqn. S1) was also varied as a parameter in the grid search. Moreover, Sect. S2 of the SI for Ref. [2] shows that CRDS-measured extinction cross-sections are biased to lower values by particle motion driven by the time-varying AC voltages of the linear electrodynamic quadrupole (LEQ) trap.[5] This low biasing can lead to inaccuracies in the retrieved real and imaginary components of the complex refractive index, which are mitigated by treating w_0 as a variable in the grid search. Here, w_0 was varied from 250 to 270 μm in 0.1 μm intervals; the beam waist calculated from geometric optics considerations is 251.3 μm, for a symmetric resonator for which the cavity length is 0.8 m, the wavelength of the intracavity beam is 405 nm, and mirror radius of curvature is 1 m. The best-fit values of w_0 retrieved from our fitting algorithm are slightly larger than this calculated value because of the aforementioned low biasing of the extinction cross-sections. Finally, a multiplicative correction factor was applied to the retrieved particle radius to account for any

bias in the retrieved particle size, with its value varied as a parameter in the grid search from 0.9900 to 1.0100 in 0.0001 intervals. The input values of n, k, w_0 and a radius correction factor that corresponded to the minimum value in χ defined the best-fit values.

S1.2 Particle Complex Refractive Index Retrievals using SP-CRDS

The algorithm described above was used to retrieve the real and imaginary components of the complex refractive index from the particle size-dependent extinction cross-sections measured using SP-CRDS for each of the four studied aqueous particles containing 4-nitrocatechol. Figure S1 depicts the contour plots corresponding to the particle size-dependent variations in extinction cross-section for the aqueous particles containing 4-nitrocatechol shown in Fig. 2 of the main manuscript (i.e., datasets for which the contour plots are not included in the main manuscript). The contour plots show the variation in the merit function with n and k, for optimized values of w_0 and the radius correction factor. Figures S1(a), (b) and (c) correspond to particles levitated in 84.3, 79.9, and 74.6% relative humidity (RH) environments (i.e., the particle size-dependent extinction cross-sections shown in Fig. 2(a), (b) and (c) of the main manuscript), respectively. The optimized values of w_0 and the radius correction factor are 257.4 μm and 0.9995, 258.0 μm and 0.9999, and 260.1 μm and 0.9998 for Fig. S1(a), (b) and (c), respectively.

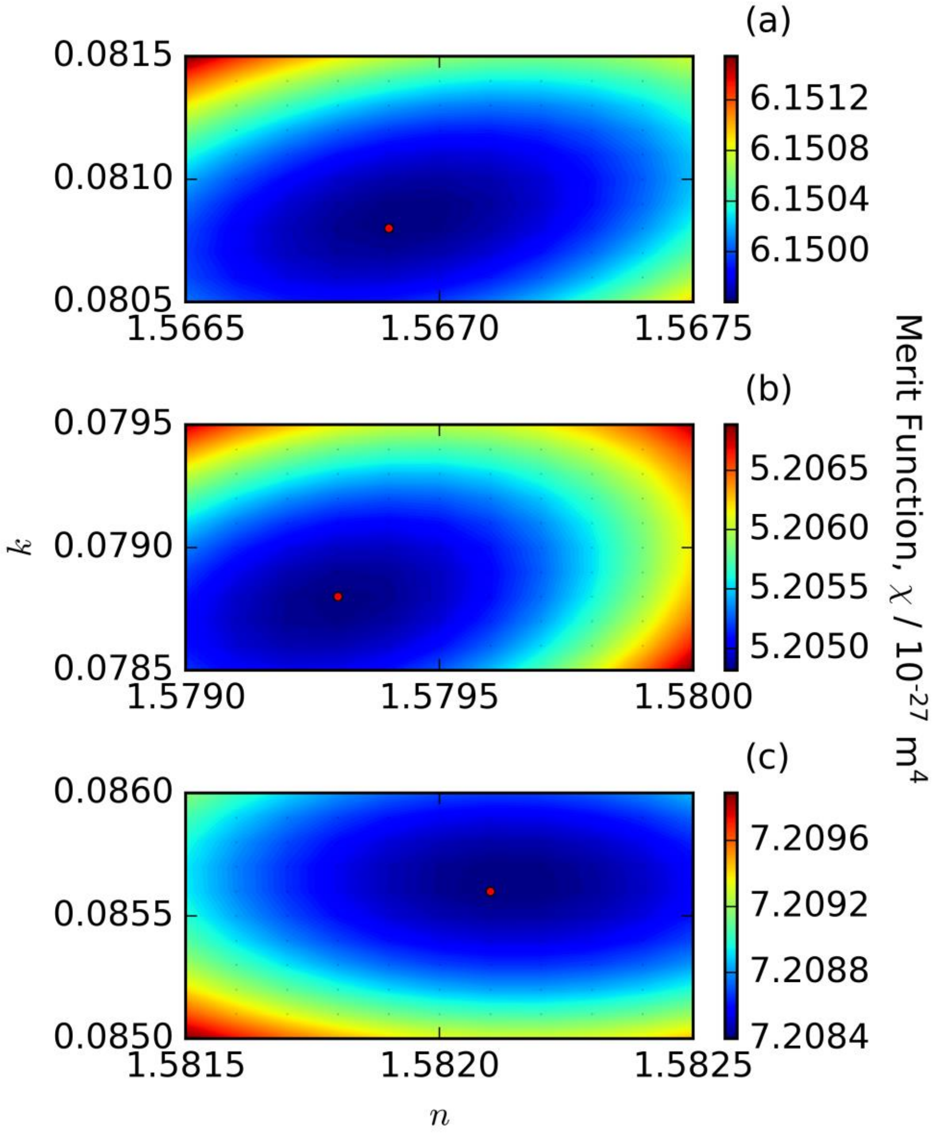

Figure S1. Contour plots depicting the variation in the merit function with n and k, for optimized values of w_0 and the radius correction factor (see main text). Panels (a), (b) and (c) correspond to the particle size-dependent extinction cross-sections depicted in Fig. 2(a), (b) and (c) of the main manuscript, respectively. The red dots indicate the minimum in the retrieved merit functions.

S1.3 Accuracy and Precision in Complex Refractive Index Retrievals using SP-CRDS

The accuracy of our aerosol particle complex refractive index retrievals using SP-CRDS measurements is assessed here using theoretical accuracy assessments that follow the same methodology as those reported in Ref. [2]. Synthetic datasets of particle size-dependent σ_{ext} were generated that represent our SP-CRDS experimental measurements for the studied aqueous particles containing 4-nitrocatechol. The assessments presented here accounted for both the smaller and slower change in particle size (with a lower limit of ~250 nm in radius

change) compared to our previous experiments.[1, 2] Inversion of these synthetic datasets using the same methods we apply to the experimental data reveal the accuracy and precision in our retrieved *n* and *k* values.

The synthetic datasets were generated assuming that the particles were comprised of water and a single solute species. The temporal evolution of particle size for steady-state evaporation in the continuum regime (i.e., the size range pertaining to the particles levitated our experiments) is given by:

$$\frac{da^2}{dt} = \frac{2M_{4\mathrm{NC}}D_{4\mathrm{NC}}}{\rho_a RT} p_{4\mathrm{NC}}\gamma_{4\mathrm{NC}}x_{4\mathrm{NC}} \times \left[\frac{(1 - x_{4\mathrm{NC}})M_{\mathrm{w}}}{x_{4\mathrm{NC}}M_{4\mathrm{NC}}} + 1\right] \quad \text{(S3)}$$

where a is the particle radius, M_{w} and $M_{4\mathrm{NC}}$ are the molar masses of water and 4-nitrocatechol, respectively, $D_{4\mathrm{NC}}$ is the gas diffusion coefficient of 4-nitrocatechol (determined by Lydersen's group contribution method),[6] R is the gas constant, T is temperature, ρ_{a} is the density of the particle, $p_{4\mathrm{NC}}$ is the vapor pressure of 4-nitrocatechol, $\gamma_{4\mathrm{NC}}$ is the activity coefficient of 4-nitrocatechol (estimated using the Aerosol Inorganic-Organic Mixtures Functional groups Activity Coefficients model; AIOMFAC),[7] and $x_{4\mathrm{NC}}$ is the mole fraction of 4-nitrocatechol. The values assigned to these parameters were representative of the studied aqueous particles containing 4-nitrocatechol and the experimental conditions: M_{w} = 18.0 g mol^{-1}, $M_{4\mathrm{NC}}$ = 155.1 g mol^{-1}, $D_{4\mathrm{NC}}$ = 6.39×10^{-6} m^2 s^{-1}, R = 8.314 J K mol^{-1}, T = 293 K. The remaining parameters depend on the composition of the aqueous particles, which is governed by the ambient RH. The mole fraction of 4-nitrocatechol was assigned a value of 0.235. This value is the same as that calculated using the radial growth factors for aqueous particles containing 4-nitrocatechol reported by Price *et al.* for a particle levitated in an 82.5% RH environment (i.e., the mean of the values studied experimentally).[8] Then, for a particle with this composition, the particle density was given a value of 1306 kg m^{-3}, determined using the ideal mixing rule (Eqn. 7 of the main article but assuming the particles were composed of water and 4NC only, i.e., $w_{4\mathrm{NC}^-}$ = 0). At the time of writing, the density of 4-nitrocatechol has not been reported, and thus the density of 4-nitrocatechol was assumed to be the same as a similar nitroaromatic compound, 4-nitrophenol (1480 kg m^{-3}).[9, 10] The activity coefficient of 4-nitrocatechol was estimated using AIOMFAC for the particle composition described here (and at 293 K) as 0.883. Finally, the

vapor pressure used was the value determined in the following subsection for pure 4-nitrocatechol (2.33×10^{-4} kg m^{-1} s^{-2}).

Particle radii were generated at a sampling rate of 15 Hz. The particle size was simulated to the nearest nanometer (i.e., the precision of our experimental retrievals from phase function measurements) from an initial radius of 1350 nm to a final radius of 1100 nm. The synthetic SP-CRDS measurements of σ_{ext} were then generated following the methodology described in Sect. 2.2 of Ref. [2], accounting for the effect of the sensitivity of ring-down time measurements and the impact of particle motion on the accuracy and precision in the extinction cross-sections. The SP-CRDS instrument used here (including the LEQ trap) is identical to that reported in Ref. [2] (excluding the longer wavelength laser used here for elastic light scattering measurements) and thus experimental parameters (e.g., the intracavity beam waist) are the same. The complex refractive indices of each of the generated datasets were identical, assigned values of $m_{405} = 1.6 + 0.8i$ (i.e., similar values to those retrieved from our SP-CRDS experiments for aqueous particles containing 4-nitrocatechol). An example synthetic dataset is depicted in Fig. S2(a).

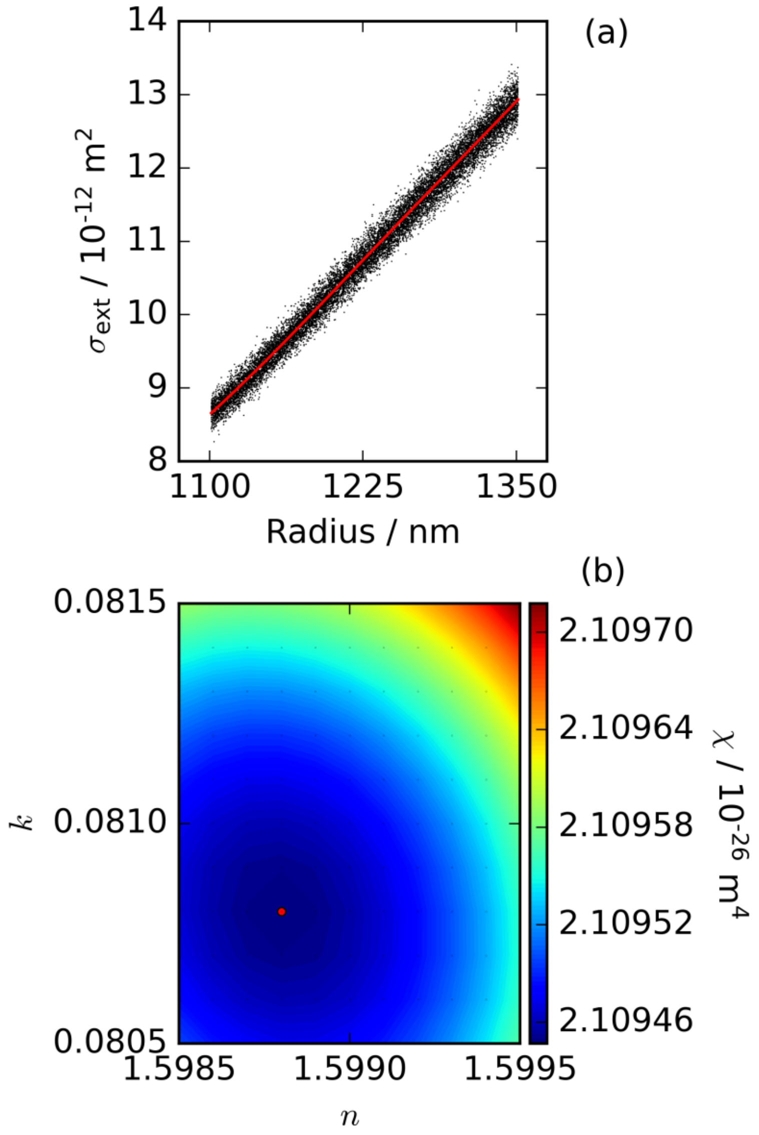

Figure S2. (a) Synthetic (black points) SP-CRDS measurements of particle size-dependent variation in extinction cross-section for an aqueous particle containing a single solute species (i.e., a model system for the aqueous particles containing 4-nitrocatechol studied here). The complex refractive index of the modelled particle was $m_{405} = 1.6 + 0.8i$. The synthetic cross-sections have been averaged to a 1 Hz sampling rate and are accompanied by the best-fit Mie theory prediction (red line). (b) The corresponding contour plot depicting the variation in the merit function (χ) with n and k, for an optimized value of w_0.

These datasets were then processed using the method of complex refractive index retrieval described in Sect. S1.1 to assess the accuracy in retrieved complex refractive indices for evaporating aqueous particles containing 4-nitrocatechol using SP-CRDS. To account for the effect of uncertainty in the particle size retrievals from phase function measurements, a + 0.1% radius bias was applied to the synthetic datasets to model any residual uncertainty in retrieved

particle size (i.e., the radius correction factor was not varied as a parameter in the grid search); see Ref. [2] for justification of the value of the applied radius bias. Figure S2(a) shows the best-fit LMT predicted extinction cross-sections, and Fig. S2(b) presents the corresponding contour plot depicting the variation in the merit function (χ) with n and k, for an optimized value of w_0 of 260.9 μm. The absolute differences between the actual (input to generate the synthetic datasets) and retrieved real and imaginary components of the complex refractive index for each of ten repeat synthetic datasets generated are depicted in Fig. S3. By repeating these simulations for ten datasets to incorporate the effects of the random nature of noise superposition on our measurements, the precision as well as the accuracy of refractive index retrievals are assessed. The mean absolute difference between the input and retrieved real refractive indices was 0.0009 $\pm$ 0.0005. The corresponding value for the imaginary refractive indices was 0.0008 $\pm$ 0.0005. The uncertainties reported represent the standard deviations in the retrieved values for the real or imaginary refractive index.

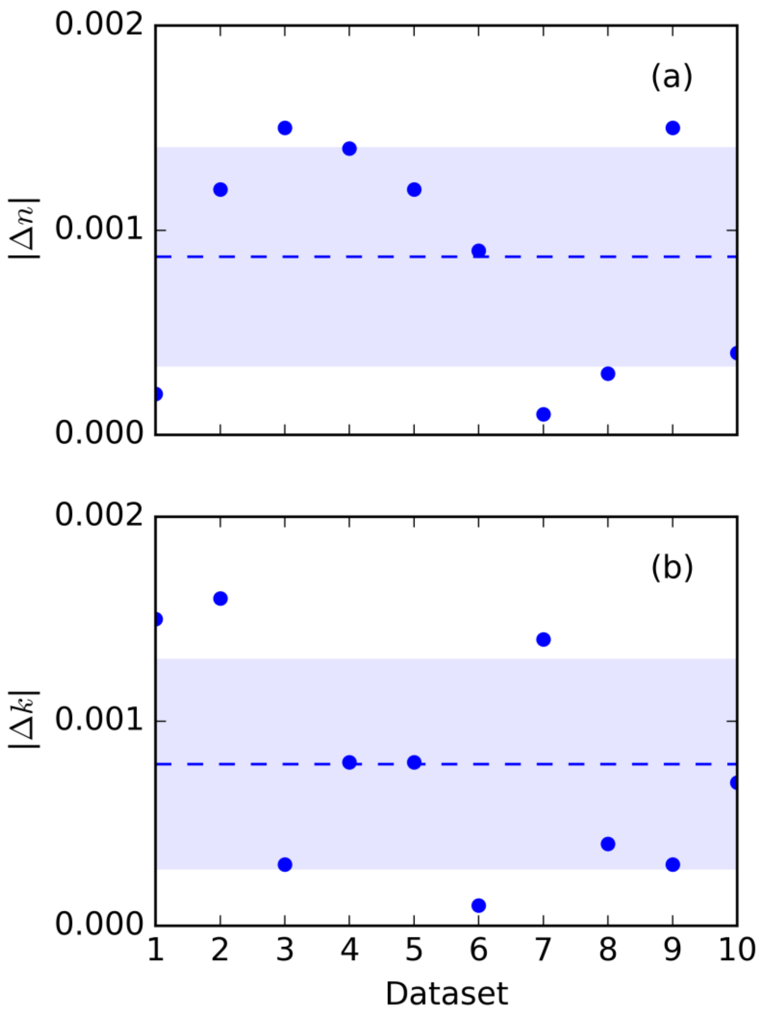

Figure S3. The absolute errors in the retrieved (a) real and (b) imaginary components of the complex refractive index from a fit of LMT predictions to the synthetic SP-CRDS measurements of particle size-dependent extinction cross-sections. Ten different synthetic datasets were generated and analyzed, each of which used a constant (i.e., invariant with particle size) complex refractive index of $m_{405} = 1.6 + 0.8i$. The dashed lines and shaded regions represent the mean and standard deviations in the retrieved values for the real or imaginary refractive index, respectively.

S1.4 The Vapor Pressure of 4-Nitrocatechol

The vapor pressures of brown carbon compounds are important quantities when considering their radiative effect because they impact the partitioning of the organic species between the particle and gas phase. The aqueous particles containing 4-nitrocatechol studied here evaporated steadily over time, with the loss of each organic molecule associated with the evaporation of water molecules concomitant with the hygroscopicity of the particle at a given RH. Therefore, the temporal evolution of particle size is given by Eqn. S3. This equation can

be rearranged straightforwardly such that the vapor pressure of 4-nitrocatechol, p_{4NC}, is the subject. The temporal evolution in the particle radius is presented in Fig. S4 for each of the four studied aqueous particles containing 4-nitrocatechol.

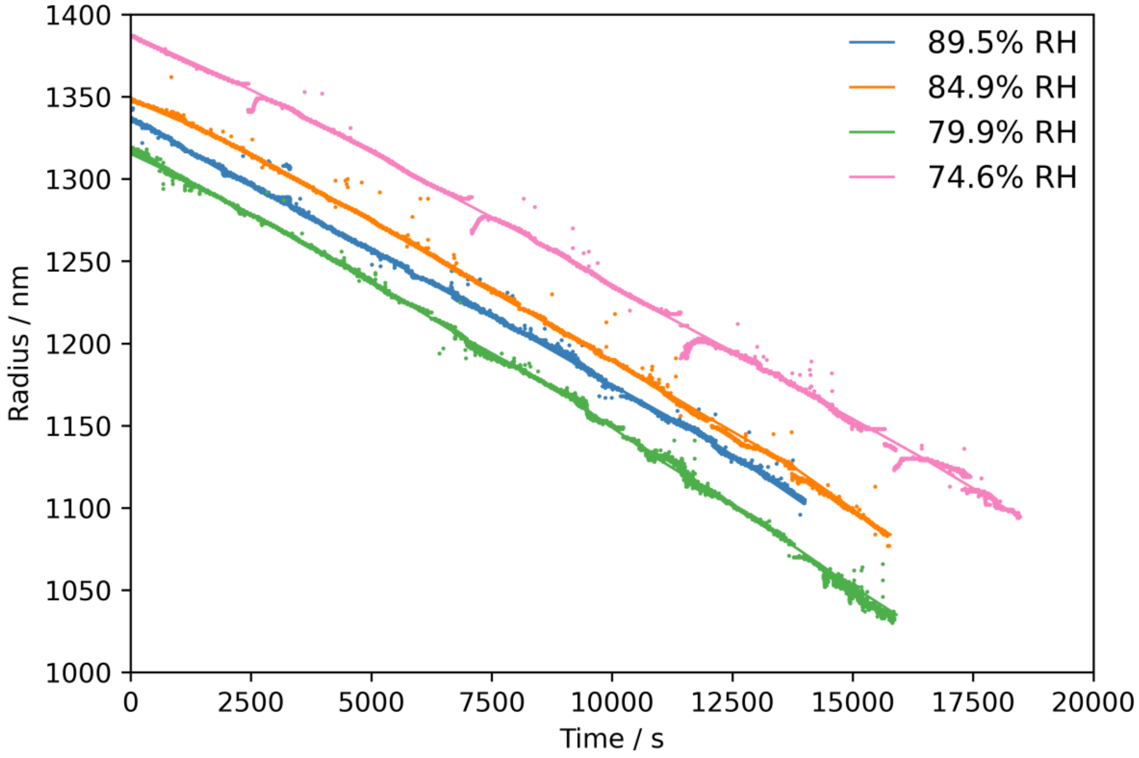

Figure S4. Temporal evolution in the particle radius for each of the four studied aqueous particles containing 4-nitrocatechol (levitated in different RH environments), determined using elastic light scattering measurements. The raw data (shown by points) have been interpolated to reduce the impact of anomalous measurements on the subsequent analysis.

The vapor pressure of 4-nitrocatechol was estimated using each of the four particles studied using SP-CRDS. The particle composition independent parameters used here are the same as those used in the previous subsection; again, assuming that the particles were composed of water and a single solute species. For each particle the composition-dependent parameters were determined. The mole fractions of 4-nitrocatechol, for each of the particles studied, were calculated using the radial growth factors for aqueous particles containing 4-nitrocatechol reported by Price *et al.*[8] The density of each particle was estimated using the ideal mixing rule (Eqn. 7 of the main article but assuming the particles were composed of water and 4NC only i.e., $w_{4\mathrm{NC}^-} = 0$), with the density of 4-nitrocatechol assumed to be the same as 4-nitrophenol (see above). Finally, the activity coefficient for 4-nitrocatechol was estimated for each particle using AIOMFAC. The values of these composition dependent parameters are summarized in

Table S1 for each particle. The mean liquid-state vapor pressure calculated for the four studied particles was 2.33 ± 0.19 × 10^{-4} Pa, where the uncertainty represents the standard deviation in the calculated vapor pressures.

Table S1. Summary of the parameters describing the experimental conditions and therefore the particle composition specific to each SP-CRDS experiment. The calculated vapor pressures of 4-nitrocatechol for each of the particles are also provided. The activity coefficients were estimated using AIOMFAC, which required input of the mole fraction of 4-nitrocatechol and the ambient temperature that was assigned a value of 293 K for each experiment.

Ambient relative humidity / %	Mole fraction of 4-nitrocatechol	Particle density / kg m^{-3}	Activity coefficient of 4-nitrocatechol	Vapor pressure of 4-nitrocatechol / 10^{-4} Pa
89.5	0.198	1281	0.863	2.53
84.3	0.021	1298	0.876	2.43
79.9	0.243	1311	0.886	2.32
74.6	0.273	1328	0.899	2.02

S1.5 Uncertainty in Real Refractive Index Retrievals using Elastic Light Scattering

Our previous determinations of the real refractive index from elastic light scattering measurements for aqueous particles containing one of a range of inorganic compounds show that the uncertainty increases with the wavelength of the illumination beam; the number of fringes present in a phase function for a fixed angular range typically decreases for smaller particle size parameters ($x = 2\pi r/\lambda$). The standard deviations in the retrieved real refractive indices were 0.004 and 0.009 for illumination wavelengths of 473 and 532 nm, respectively.[11] Therefore, we assign a value of 0.01 to the uncertainty in the real refractive indices retrieved from our elastic light scattering measurements at a wavelength of 632.8 nm.

S2 UV/visible Spectroscopy

This section presents the UV/visible absorption spectra for aqueous solutions of 4-nitrocatechol and either HCl or NaOH (Sect. S2.1), and how these absorption spectra change with time (Sect.

S2.2). These absorption spectra are then used to determine the wavelength-dependent imaginary refractive index for 4NC, $4NC^-$ and $4NC^{2-}$ (Sect. S2.3). Section S2.4 uses a Henderson–Hasselbalch model incorporating reported pK_a values to determine the fractional composition of the 4-nitrocatechol species 4NC, $4NC^-$ and $4NC^{2-}$.

S2.1 Absorption Spectra for Aqueous Solutions of 4-Nitrocatechol

UV/visible spectroscopy was used to measure the absorption spectra for the preprepared aqueous solutions of 4-nitrocatechol (5×10^{-5} M; see Sect. 2.2 of the main manuscript), obtained at a broad range of pH values. In each case, the pH of the solution was adjusted with either HCl or NaOH. The measured spectra presented in Figure S5 show an increase in absorbance at longer (visible) wavelengths with pH.

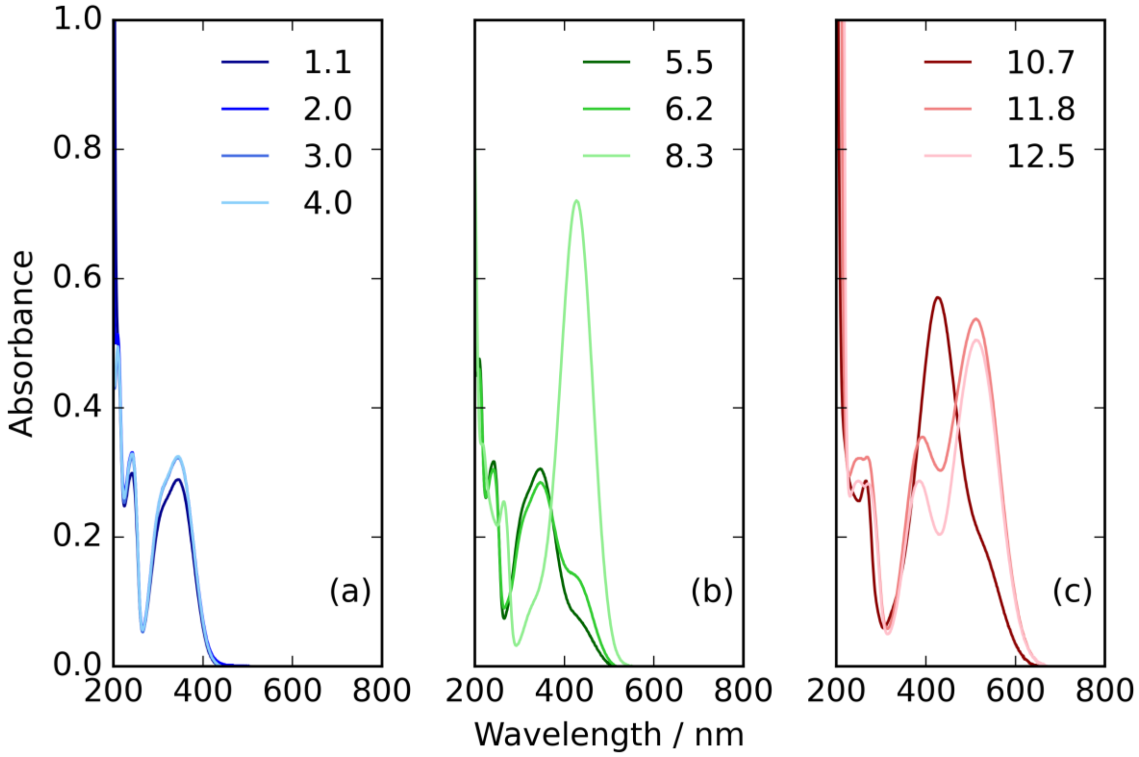

Figure S5. Absorption spectra for aqueous solutions of 4-nitrocatechol (5×10^{-5} M), measured using UV/visible spectroscopy. The pH of each solution was adjusted with either HCl or NaOH, with the values for each solution provided in the legend. The panels show spectra for solutions for which: (a) 4NC is the predominant species, (b) the equilibrium between 4NC and $4NC^-$ is progressively shifted, increasing the concentration of $4NC^-$, (c) the equilibrium between $4NC^-$ and $4NC^{2-}$ is progressively shifted, increasing the concentration of $4NC^{2-}$.

S2.2 Temporal Variation in Absorption Spectra for Aqueous Solutions of 4-Nitrocatechol

Twelve aqueous solutions of 4-nitrocatechol (5×10^{-5} M) were prepared, each with a different pH value. Again, the pH of the solution was adjusted with either HCl or NaOH. UV/visible spectroscopy was used to measure absorbance spectra for each solution within 30 min of their preparation. These spectra, and additional measurements made over the following seven days, are presented in Fig. S6. The solutions were wrapped in tinfoil and stored in a dark cupboard between measurements. The absorbance spectra measured for solutions containing singly deprotonated and doubly deprotonated forms of 4-nitrocatechol are unstable over time scales of ~24 h.

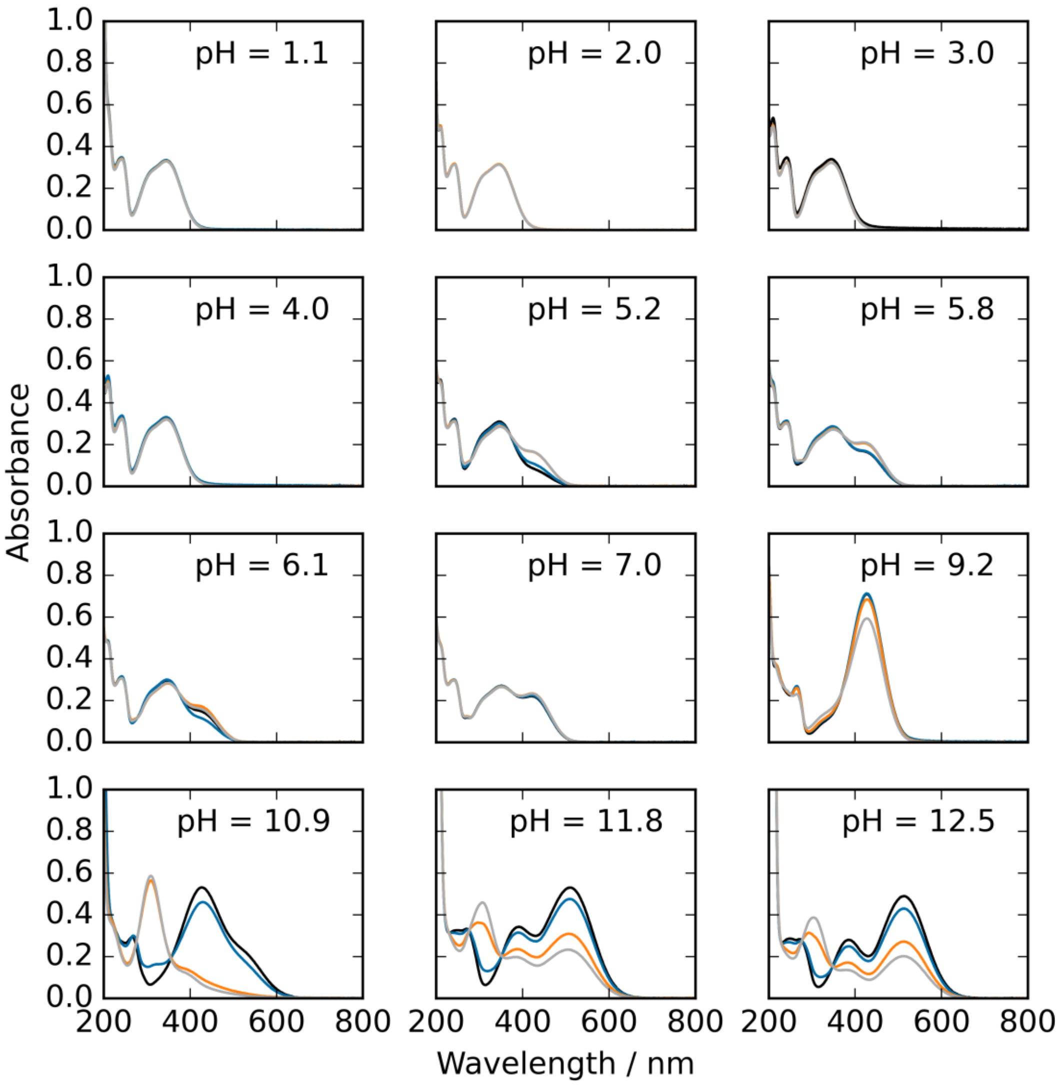

Figure S6. Absorption spectra for aqueous solutions of 4-nitrocatechol (5×10^{-5} M) for which the initial pH was adjusted with either HCl or NaOH, measured using UV/visible spectroscopy. Spectra were measured within 30 min of the preparation of the solutions (day 1) and on day 2, 4 and 7 (black, blue, orange and grey lines, respectively). The pH values for each solution measured on day 1 are provided in the panel label.

S2.3 Wavelength-Dependent Imaginary Refractive Indices for 4NC, $4NC^-$ and $4NC^{2-}$

The wavelength-dependent imaginary refractive indices for the aqueous solutions of 4-nitrocatechol were determined from the measured absorption spectra using Eqn. 1 of the main manuscript. Then, using a linear weighting of the imaginary refractive indices of mixed components by their mass fractions (see Sect. 3.2 of the main manuscript), the imaginary refractive index values were determined for the pure component 4NC, $4NC^-$ and $4NC^{2-}$ species. The wavelength-dependent imaginary refractive index for the pure 4NC component was calculated from the imaginary refractive index spectra for an aqueous solution of 4-nitrocatechol and HCl with pH = 1.1 (shown in Fig S5(a)), in which the concentrations of $4NC^-$ and $4NC^{2-}$ are negligible, according to:

$$k(\lambda) = k_{\mathrm{4NC}}(\lambda)\mathrm{w}_{\mathrm{4NC}} \qquad \text{(S4)}$$

The wavelength-dependent imaginary refractive index for the pure $4NC^-$ component was determined using a UV/visible absorption spectrum for an aqueous solution of 4-nitrocatechol and NaOH with pH = 8.3 (shown in Fig. S5(b)). Figure 4 of the main manuscript indicates that a solution with this pH contains a negligible fraction of $4NC^{2-}$ (<0.3%), i.e., an order of magnitude less than the fraction of 4NC present within the solutions. Therefore, the wavelength-dependent imaginary refractive index of $4NC^-$ was given by:

$$k(\lambda)_{\mathrm{4NC^-}} = \frac{k(\lambda) - k(\lambda)_{\mathrm{4NC}}\mathrm{w}_{\mathrm{4NC}}}{\mathrm{w}_{\mathrm{4NC^-}}} \qquad \text{(S5)}$$

Similarly, the wavelength-dependent imaginary refractive index of the pure $4NC^{2-}$ component was estimated from the UV/visible absorption spectrum for an aqueous solution of 4-nitrocatechol and NaOH with pH = 12.5 (see Fig. S5(c)). The wavelength-dependent imaginary refractive index of $4NC^{2-}$ was determined by solving:

$$k(\lambda)_{\mathrm{4NC^{2-}}} = \frac{k(\lambda) - (k(\lambda)_{\mathrm{4NC}}\mathrm{w}_{\mathrm{4NC}} + k(\lambda)_{\mathrm{4NC^-}}\mathrm{w}_{\mathrm{4NC^-}})}{\mathrm{w}_{\mathrm{4NC^{2-}}}} \qquad \text{(S6)}$$

S2.4 Fractional Composition of 4-Nitrocatechol Species Determined using a Henderson–Hasselbalch model

In the following analysis for dilute aqueous solutions containing 4-nitrocatechol solute, we assume that the activity coefficients for H^+ and the solute species are equal to unity, as these activity coefficients are not measurable directly and are challenging to predict with accuracy. The fractional composition of 4-nitrocatechol, i.e., the fractions of protonated (α_1), singly deprotonated (α_2) and doubly deprotonated (α_3) 4-nitrocatechol within the aqueous solutions of 4-nitrocatechol, were determined using a Henderson–Hasselbalch model, derived from the acid dissociation constant (defined by Eqn. S7), incorporating reported pK_a values for the equilibria.[12] As written, the equation assumes that there are no non-ideal molecular interactions, an appropriate assumption for the dilute aqueous solutions of 4-nitrocatechol studied in this section.

$$K_a = \frac{[H^+][A^-]}{[HA]} \tag{S7}$$

For a diprotic acid, such as 4NC, there are two acid dissociation constants (one for each acidic proton):

$$K_{a1} = \frac{[H^+][HA^-]}{[H_2A]} \tag{S8}$$

$$K_{a2} = \frac{[H^+][A^{2-}]}{[HA^-]} \tag{S9}$$

The concentration of each of the conjugate base species can therefore be expressed as:

$$[HA^-] = K_{a1}\frac{[H_2A]}{[H^+]} \tag{S10}$$

$$[A^{2-}] = K_{a2}\frac{[HA^-]}{[H^+]} = K_{a1}K_{a2}\frac{[H_2A]}{[H^+]^2} \quad (S11)$$

The fraction of the acid can then be calculated according to:

$$\alpha_1 = \frac{[H_2A]}{[H_2A] + [HA^-] + [A^{2-}]} = \frac{[H^+]^2}{[H^+]^2 + K_{a1}[H^+] + K_{a1}K_{a2}} \quad (S12)$$

This methodology can be repeated to formulate equations for the fractions of the singly and doubly deprotonated species:

$$\alpha_2 = \frac{K_{a1}[H^+]}{[H^+]^2 + K_{a1}[H^+] + K_{a1}K_{a2}} \quad (S13)$$

$$\alpha_3 = \frac{K_{a1}K_{a2}}{[H^+]^2 + K_{a1}[H^+] + K_{a1}K_{a2}} \quad (S14)$$

Cornard *et al.*, reported that the pK_a values of 4NC and $4NC^-$ are pK_{a1} = 6.7 and pK_{a2} = 10.8, with $K_a = 10^{-pK_a}$. The concentration of H^+ was calculated as a function of solution pH using Eqn. S15.

$$pH = -\log_{10}([H^+]) \quad (S15)$$

Figure 4 of the main article shows the fractions of 4NC, $4NC^-$ and $4NC^{2-}$ present in solution, as a function of solution pH, predicted using Eqns. S12-S14.

S3 Critical Point Model

The critical point model describes the optical transitions of a species as the sum of a set of critical point line shapes. Section S3.1 determines the fractional composition of 4-nitrocatechol species within our aqueous solutions using these critical point line shapes, applying a spectral decomposition algorithm to our measured absorption spectra (see Fig. S5). These measured

fractional compositions validate the pK_a values used by the Henderson–Hasselbalch model in the previous section.

The critical point model is used in the main manuscript to estimate the wavelength-dependent real refractive index for pure 4NC and $4NC^-$ from the corresponding imaginary refractive index distributions. Each of these inversions were constrained using the real refractive index of the pure component species determined at a single wavelength. To determine the real refractive index of the pure component species, we first had to estimate the difference between the real refractive index of the 4NC and $4NC^-$ at these single wavelengths (Sect. S3.2). The parameters pertaining to the critical point lineshapes used to describe the pure component wavelength-dependent refractive indices for 4NC and $4NC^-$ are provided in Sect. S3.3. The uncertainty in the estimated pure component wavelength-dependent real refractive indices for 4NC and $4NC^-$ are discussed in Sect. S3.3.

S3.1 Fractional Composition of 4-Nitrocatechol Species Determined Using a Spectral Decomposition Algorithm

Figure 4 of the main manuscript shows the predicted pH-dependent fractions of 4NC, $4NC^-$ and $4NC^{2-}$ from a Henderson–Hasselbalch model incorporating pK_a values reported by Cornard *et al.* for their equilibria.[12] These pK_a values were tested by estimating the fraction of species within our own aqueous solutions of 4-nitrocatechol.

UV/visible absorption spectra for each of the different pure forms of 4-nitrocatechol (Fig. 5 of the main manuscript) were fit using a sum of *l* critical point oscillators of the form:[13]

$$\alpha_l = \frac{A_l}{\lambda_l}\left[\frac{e^{i\phi_l}}{\frac{1}{\lambda_l}-\frac{1}{\lambda}-\frac{i}{\gamma_l}} + \frac{e^{-i\phi_l}}{\frac{1}{\lambda_l}+\frac{1}{\lambda}+\frac{i}{\gamma_l}}\right] \tag{S16}$$

in which α_l is the contribution of the *l*th oscillator to the absorption, A_l corresponds approximately to an amplitude, λ_l to a centre wavelength, γ_l to a linewidth and ϕ_l is a phase factor. Each species was fit to four or five oscillators as appropriate to capture the spectrum accurately. We note that the analysis in the main manuscript used six oscillators to reproduce measured UV/visible spectra, with slight improvement compared to when using < 6 oscillators. However, we found 4-5 oscillators to allow for robust determinations of fractional

contributions to spectra from the three protonation states of 4NC. The oscillator parameters determined for each of the pure species were then used to determine their concentrations in a mixture. For mixtures, we first identify the species present by visual inspection of the spectrum. We then fit the spectrum of the mixture using the oscillator parameters for the relevant pure species. For each form, the parameters λ_l, γ_l and ϕ_l are fixed, but the amplitudes A_l are allowed to vary to reproduce the measured spectrum. We then calculate the ratio A_l^{mix}/A_l^{pure}, where A_l^{mix} is the amplitude of the oscillator in the mixture and A_l^{pure} is the amplitude in the pure spectrum, for each of the oscillators of a species and take an average to determine the fractional concentration. These measured fractions of each 4-nitrocatechol species are represented by the points in Fig. 4 of the manuscript.

S3.2 Differences Between the Wavelength-Dependent Real Refractive Indices for 4NC and $4NC^-$

In Sect. 3.3.2 of the main manuscript, the pure component real refractive indices of 4NC and $4NC^-$ were determined at 405- and 632.8-nm from our SP-CRDS and elastic light scattering measurements, respectively, for aqueous particles containing 4-nitrocatechol. To estimate these pure component values, we first found the difference between the real refractive index of the two species at each wavelength (see Sect. 3.3.2). Then, the critical point model was used to reveal the wavelength-dependent real refractive index distributions (see Sect. 3.3.1 for details of the critical point model). Figure S7(a) shows the wavelength-dependent imaginary refractive indices for 4NC (blue lines) and $4NC^-$ (green dashed lines) obtained from a fit of the critical point model predictions to the measured distributions (black dashed lines). The critical point model is KK consistent and therefore allowed the corresponding wavelength-dependent real refractive index distributions for 4NC (blue line) and $4NC^-$ (green line) to be determined, depicted in Fig. S7(b). The inversions were constrained by setting the values of the real refractive index of both 4NC and $4NC^-$ to be equal to unity at a wavelength of 1 mm (see main text for details). From this analysis, we estimated the difference between the real refractive indices of 4NC and $4NC^-$ at wavelengths of 405 and 632.8 nm to be 0.482 and 0.061, respectively. Note that the real refractive index values in Figure S7 are not final estimates of the true real refractive index values but instead represent offset relative distributions in the real refractive index that are corrected by the analysis presented in Sect. 3.3.2 of the main manuscript. For clarity, these relative real refractive index values are labelled n_{rel}.

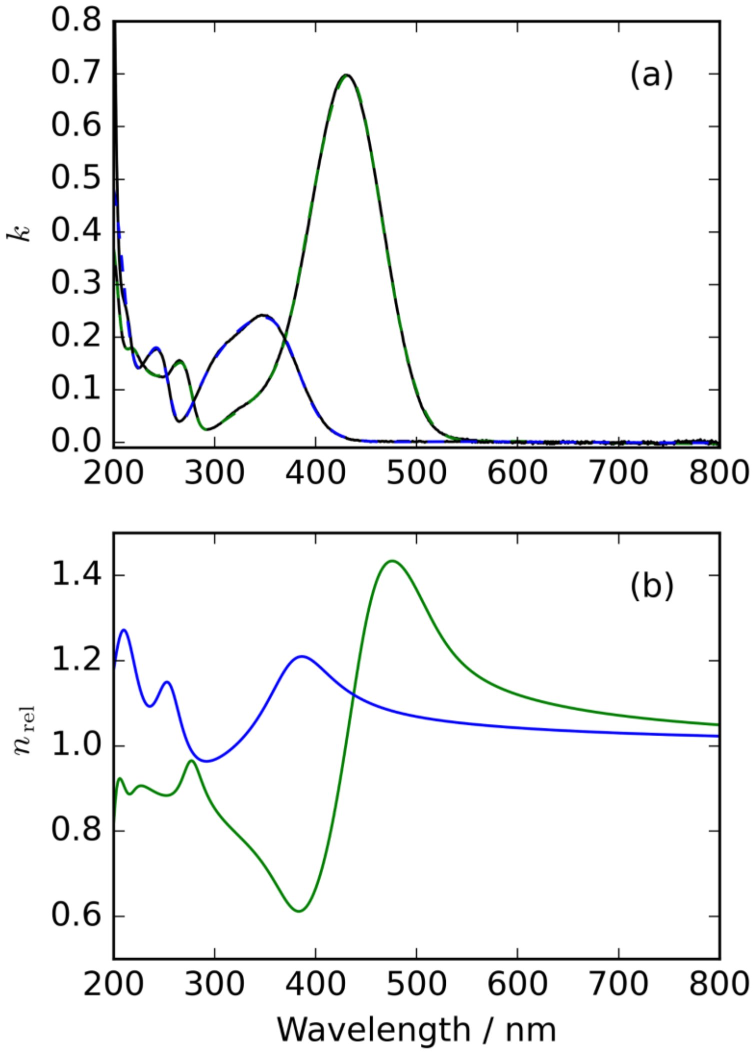

Figure S7. Kramers-Kronig analysis of the imaginary and real refractive indices of pure component 4-nitrocatechol species. (a) The wavelength-dependent imaginary refractive index for 4NC (blue dashed lines) and $4NC^-$ (green dashed lines) obtained from a fit of critical point model predictions to the measured distributions (black lines). (b) (b) The corresponding relative distribution in the real refractive index distributions for 4NC (blue line) and $4NC^-$ (green line) determined from the KK relationship. The inversions were constrained by setting the values of the real refractive index of both 4NC and $4NC^-$ to be equal to unity at a wavelength of 1 mm.

S3.3 Critical Point Lineshapes Describing the Wavelength-Dependent Refractive Index for Pure Component 4NC and $4NC^-$

The critical point model describes the optical transitions of a species as the sum of a set of critical point line shapes to model the wavelength-depended refractive index. Each lineshape is described by four parameters: A_l is the amplitude of the contribution from oscillator l, λ_l is the resonance wavelength, γ_l is a broadening parameter, and ϕ_l denotes a phase factor that

allows for an asymmetric line shape. Additionally, ϵ_0 is a constant that accounts for contributions to the refractive index from optical transitions at wavelengths shorter than the wavelength range studied here.

Figure 6 of the main manuscript depicts the wavelength-dependent real and imaginary refractive indices for pure 4NC and $4NC^-$ determined using the critical point model. The critical point model was constrained using real refractive index values at a single wavelength determined using either 405-nm single particle CRDS or elastic light scattering measurements at a wavelength of 632.8 nm. The values of the parameters describing each of the critical point lineshapes for pure 4NC determined using the critical point model constrained using a single real refractive index value at either 405- or 632.8-nm are reported in Table S2 and S3, respectively.

Table S2. Parameter values pertaining to the six critical point lineshapes (i.e., up to l=6) used to describe the pure component wavelength-dependent refractive index for 4NC. The critical point model was constrained using a single real refractive index value at 405-nm determined using single particle CRDS. The best-fit value of ϵ_0 was 2.137.

	A_l / unitless	λ_l / nm	γ_l / nm	ϕ_l / radians
$l = 1$	0.0913	498.7	4292	3.073
$l = 2$	0.3043	448.5	4212	-0.208
$l = 3$	0.0189	373.7	3977	2.671
$l = 4$	0.0192	276.5	6001	-1.269
$l = 5$	0.0079	219.1	6620	0.560
$l = 6$	0.0366	200.4	4604	0.096

Table S3. Parameter values pertaining to the six critical point lineshapes (i.e., up to l=6) used to describe the pure component wavelength-dependent refractive index for 4NC. The critical point model was constrained using a single real refractive index value at 632.8-nm determined using elastic light scattering measurements. The best-fit value of ϵ_0 was 2.281.

	A_l / unitless	λ_l / nm	γ_l / nm	ϕ_l / radians
$l = 1$	0.1014	498.7	4347	3.073
$l = 2$	0.3333	449.2	4006	-0.215
$l = 3$	0.0278	371.8	3344	2.845
$l = 4$	0.0200	276.2	5844	-1.311
$l = 5$	0.0119	219.1	4663	0.422
$l = 6$	0.0299	200.4	6174	0.164

The corresponding values describing each of the critical point lineshapes for pure $4NC^-$ are reported in Table S4 and S5, respectively.

Table S4. Parameter values pertaining to the four critical point lineshapes (i.e., up to l=4) used to describe the pure component wavelength-dependent refractive index for $4NC^-$. The critical point model was constrained using a single real refractive index value at 405-nm determined using single particle CRDS. The best-fit value of ϵ_0 was 1.907.

	A_l / unitless	λ_l / nm	γ_l / nm	ϕ_l / radians
$l = 1$	0.0736	381.9	3669	-1.357
$l = 2$	0.3422	322.3	713	-0.207
$l = 3$	0.0521	254.7	3864	-1.640
$l = 4$	0.1139	210.0	2616	-1.348

Table S5. Parameter values pertaining to the four critical point lineshapes (i.e., up to l=4) used to describe the pure component wavelength-dependent refractive index for $4NC^-$. The critical point model was constrained using a single real refractive index value at 632.8-nm determined using elastic light scattering measurements. The best-fit value of ϵ_0 was 1.899.

	A_l / unitless	λ_l / nm	γ_l / nm	ϕ_l / radians
$l = 1$	0.0784	381.3	3608	-1.334
$l = 2$	0.4468	320.3	611	-0.038
$l = 3$	0.0546	255.5	3842	-1.733
$l = 4$	0.1403	210.0	2436	-1.475

S3.4 Uncertainty in the Real Refractive Indices for 4NC and $4NC^-$ Estimated from SP-CRDS and Elastic Light Scattering Measurements

In Sect. 3.3 of the main manuscript, the pure component real refractive indices for 4NC and $4NC^-$ were estimated at a wavelength of 405 nm from the SP-CRDS-determined real refractive index values for aqueous particles containing 4-nitrocatechol. The mean values of the real components of the complex refractive indices for 4NC and $4NC^-$ obtained from all four particles were 1.797 ± 0.017 and 1.313 ± 0.017, respectively. The accuracies of the pure component real refractive indices were estimated from propagation of uncertainties in the real refractive index determined from SP-CRDS measurements, the fractions of 4NC and $4NC^-$ within the particles estimated in Sect. 3.2 of the main manuscript, and the radial growth factors reported by Price *et al.*[8] The accuracies in the aerosol particle real refractive indices retrieved from SP-CRDS measurements were determined to be 0.0014 (i.e., the mean absolute difference between the input and retrieved real refractive indices in our analysis plus one standard deviation; see Sect. S1.5). The uncertainty in the fractions of 4NC and $4NC^-$ within the particles of 0.4% (see Sect. 3.2 of the main manuscript) leads to an uncertainty in their real refractive indices of <0.0005 and thus has a negligible effect on the determined refractive indices. Finally, the radial growth factors reported by Price *et al.* have typical uncertainties of ~0.01 and therefore the accuracy of the radial growth factors is assigned a value of 0.01.

Similarly, the pure component real refractive indices for 4NC and $4NC^-$ were estimated at a wavelength of 632.8 nm from the real refractive index values for aqueous particles containing 4-nitrocatechol determined using elastic light scattering measurements (see Sect. 3.3 of the main manuscript). The mean values of the pure component real refractive indices from all four

particles at 632.8-nm were 1.703 ± 0.031 and 1.765 ± 0.031 for 4NC and $4NC^-$, respectively. The accuracies of the pure component real refractive indices at 632.8-nm were estimated from propagation of uncertainties in the real refractive index determined from elastic light scattering measurements, the fractions of 4NC and $4NC^-$ estimated in Sect. 3.2 of the main manuscript, and the radial growth factors reported by Price *et al.* For the reasons described in Sect. S1.5, we assigned a value of 0.01 for the uncertainty in the real refractive indices retrieved from our elastic light scattering measurements.

The uncertainties in the determined pure component real refractive indices for 4NC and $4NC^-$ at both 405- and 632.8-nm for the four distinct aqueous particles containing 4-nitrocatechol studied here vary by ~10% between particles because of the different particle compositions for the particles held in different RH environments; the different contributions to the calculated uncertainty from propagation of error carry different weights depending on the particle composition, and therefore the RH. The uncertainties stated above correspond to the largest uncertainty values estimated for all four particles, such that our estimates are conservative.

S4 The Influence of Illumination Beam Wavelength on the Temporal Evolution in Particle Size

This section presents measurements of the rate of change in particle size for aqueous particles containing 4-nitrocatechol illuminated with the unfocused output beam from either a 532- or 632.8-nm laser. If the particle is light-absorbing at the wavelength of the incident beam, then particle heating will occur. Increasing the temperature of the particle causes water to partition from the particle into the gas phase to maintain the equilibrium of the particle water activity with the RH surrounding the particle, increasing the rate of change in particle radius. The motivation for these experiments was two-fold. Firstly, light-induced heating perturbs the composition of the studied particles, and thus the particle properties, including the size and refractive index. Not heating the aerosol particles allowed their composition to be determined using the literature values for the radial growth factor of aqueous particles containing 4-nitrocatechol (confirmed not to be affected by light-induced heating).[8] It should be noted that these authors did not determine the pH of their particles. However, differences in the pH of the particles studied in their experiments and here were expected to be negligible because of the comparable size and composition of the particles. Second, the particle heating, or lack thereof, provides an indication of the composition of the studied particles (see below).

The rate of change in particle size was determined for aqueous particles containing 4-nitrocatechol, while ensuring that the RH surrounding the particles was the same for each experiment (~88%). The particles were either illuminated continuously throughout the experiment or intermittently for short periods of time (for 30 s at a time, at time intervals of 15 min). The experiments for which the particle was illuminated intermittently were used as a model system to determine the rate of change in particle size for an unperturbed (not illuminated) particle. Figure S8 depicts the reduction in the square of the particle radius with time, with all values normalized to the square of the radius at the start of the measurement.

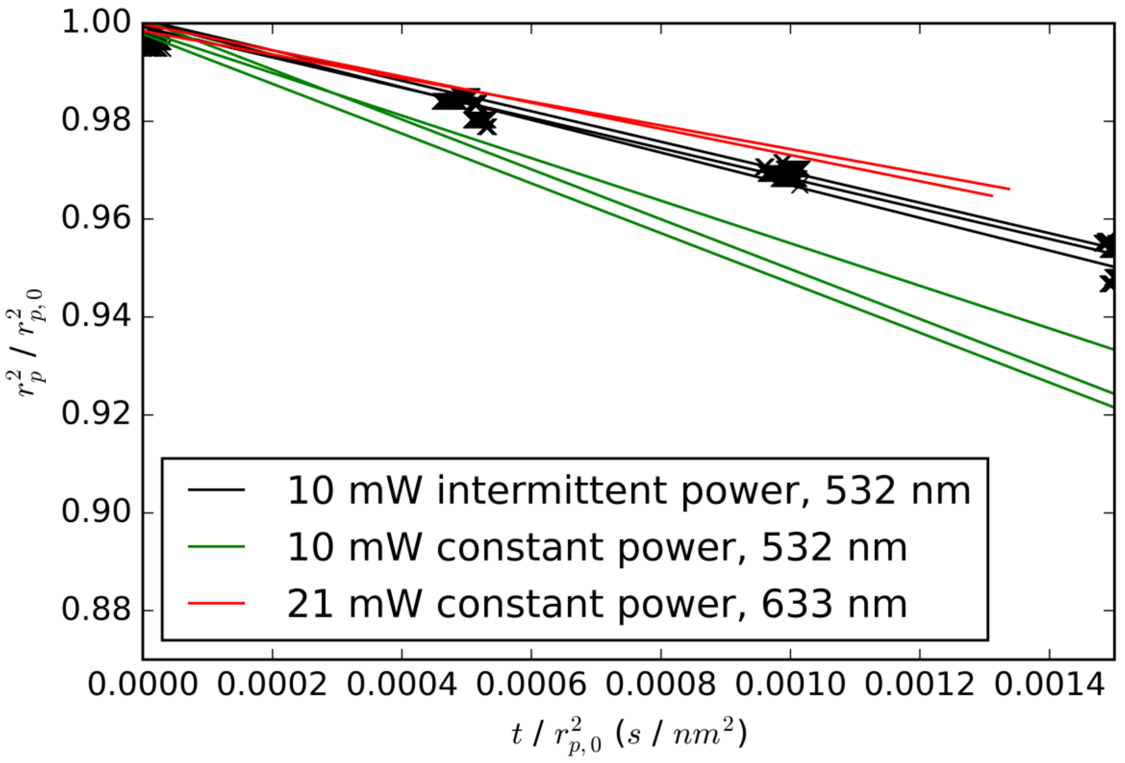

Figure S8. Reduction in size of aqueous particles containing 4-nitrocatechol illuminated by 532-nm or 632.8-nm laser beams under different conditions of laser power or irradiation method. Lines of best-fit (calculated using the least squares method) to the square of the radius plotted as a function of time, are all normalized by the value of the square of the radius at illumination time $t = 0$. The green and red lines represent measurements for which the particles were illuminated constantly during the experiment using the 532-nm and 632.8-nm laser sources, respectively. The black lines represent measurements for which the particles were illuminated using the 532-nm laser source intermittently (see text). Black crosses indicate the times at which the particles were illuminated.

Doubly deprotonated 4-nitrocatechol is expected to absorb light strongly at long visible wavelengths (see Fig. 5 of the main manuscript). Therefore, a particle containing $4NC^{2-}$ would evaporate more rapidly on illumination with the 632.8-nm laser beam used for elastic light scattering measurements than one containing no $4NC^{2-}$. There was no difference in the rate of change in particle size for particles illuminated continuously using a 632.8-nm beam or intermittently using a 532-nm beam (i.e., the model system for an unperturbed particle) that could not be attributed to small changes in the ambient conditions (e.g., changes in lab temperature of ~1 °C). Therefore, it was assumed that the studied particles did not contain $4NC^{2-}$. Conversely, the particles were expected to contain $4NC^{-}$ because aqueous particles containing 4-nitrocatechol evaporated faster when a 532-nm laser beam was used in place of the 632.8-nm laser for elastic light scattering measurements; Figure 5 of the main manuscript suggests that 4NC does not absorb 532-nm light, while $4NC^{-}$ strongly absorbs light of this wavelength.

Despite both 4NC and $4NC^{-}$ absorbing light at 405-nm, the intra-cavity CRDS beam did not contribute to particle heating because it reaches only sub-milliwatt power levels, and these power levels are transient (with durations of less than ~30 μs). Repetition rates of ~20 ring-down events per second correspond to one-second integrated power levels of ~1 μW. Additionally, we further ensured that heating effects were suppressed by keeping the power of the 632.8-nm illumination laser at a low level of ~10 mW, which proved sufficient for acquisition of phase function images.

S5 Calibration of the Capacitance Probe used for Measurements of Relative Humidity

During the SP-CRDS experiments reported in Sect. 3.1 of the main manuscript, the relative humidity surrounding the particles was recorded continuously using a capacitance probe positioned ~1 cm from the position of the studied particles. This section describes the calibration of the probe using the experimentally determined real refractive index for aqueous, non-absorbing, particles of ammonium sulfate (AS). Ammonium sulfate is an inorganic species for which the optical and hygroscopic properties are well characterized.[11, 14]

A bulk solution of aqueous AS was prepared with HPLC plus water. The solutions were loaded into a droplet-on-demand dispenser from which aerosol particles were generated and injected into the LEQ trap. After rapid equilibration of the freshly generated particles with the ambient RH, a single aerosol particle was selected. Following the method described in Sect. 2.1 of the main manuscript, particle size-dependent extinction cross-sections were measured using SP-

CRDS for aqueous particles containing AS. Here, the particle size-change was driven by controlled change in the RH, and the subsequent gas-particle partitioning of water as the water activity of the particle equilibrated with the ambient RH. The CRDS-measured size-dependent variation in extinction cross-section for an aqueous particle containing AS is depicted in Fig. S9(a), and shows two distinct features: sharp resonance structure and broad interference structure.[3, 15]

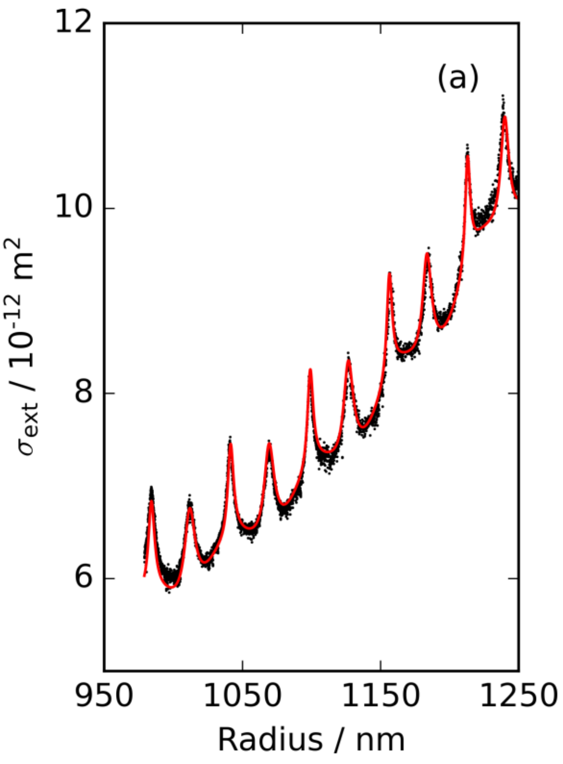

Figure S9. (a) Measured (black points) size-dependent variation in extinction cross-section, averaged to a 1 Hz sampling rate, for an aqueous particle containing AS. The best-fit LMT distribution is overlaid (red line). (b) The retrieved real refractive index is shown in red, plotted against the value of the ambient RH (as determined using the capacitance probe). The real refractive index distribution determined from a repeat measurement is provided in blue. The purple dots are the retrieved mean real refractive index values for aqueous particles containing AS determined using SP-CRDS, taken from Cotterell *et al.* (for which the capacitance probe was calibrated using an in-house calibration apparatus).[11] The purple fill represents the standard deviation in these previously retrieved real refractive index values.

The measured extinction cross-sections were averaged to 1 Hz intervals and fitted to predictions from LMT, using a differential evolution (DE) algorithm. The imaginary refractive

index for the particle was assumed to be zero at 405-nm, because AS is non-absorbing at visible wavelengths.[14] As the particle size was reduced, the involatile AS was concentrated, and thus composition of the studied particles was dependent on their size. Therefore, the size-dependent real refractive index was parameterized using the following empirical expression and input to calculations of the LMT predictions of extinction cross-section:

$$n = n_0 + \frac{n_1}{r^3} + \frac{n_2}{r^6} + \frac{n_3}{r^9} \quad \text{(S17)}$$

The parameters for each vector in the DE algorithm were n_1, n_2, n_3, the beam waist of the TEM_{00} Gaussian cavity mode at the location of the trapped particle, and a multiplicative radius correction factor. The reasons for treating the beam waist as a parameter in the DE algorithm have been described in Sect. S1.1. The radius correction factor accounted for any bias in the retrieved particle size. The value of n_0 was assigned a value of 1.3388 because, as the particle takes up water and $r \rightarrow \infty$, $n = n_0$, where n_0 corresponds to the real refractive index of pure water at a wavelength of 405 nm.[16]

The best-fit LMT prediction for the particle size-dependent variations in extinction cross-section and corresponding retrieved real refractive indices are depicted in Fig. S9(a) and (b), respectively. The uncertainty in the retrieved values is not known as there have been no formal accuracy assessments for retrievals of the particle size-dependent real refractive index using the DE algorithm. However, the agreement between the measured and modelled cross-sections indicates that the refractive index retrievals are accurate. The refractive indices retrieved here agree with those reported previously by Cotterell *et al.* for RH >70%, for which a capacitance RH probe was calibrated using an in-house calibration apparatus.[11] Therefore, the accuracy of the capacitance probe used here was assumed to be the same as that reported by Cotterell *et al.* (±2%; i.e., the standard error of the capacitance probe measurements of RH).[11]

References

(1) Knight, J. W.; Egan, J. V.; Orr-Ewing, A. J.; Cotterell, M. I. Direct Spectroscopic Quantification of the Absorption and Scattering Properties for Single Aerosol Particles. *J. Phys. Chem. A* **2022**, *126* (9), 1571-1577. DOI: 10.1021/acs.jpca.2c00532.

(2) Knight, J. W.; Orr-Ewing, A. J.; Cotterell, M. I. Evaluating the accuracy of absorbing aerosol optical properties measured using single particle cavity ring-down spectroscopy. *Aerosol Sci. Technol.* **2023**, *57* (5), 406-424. DOI: 10.1080/02786826.2023.2185500.
(3) Cotterell, M. I.; Knight, J. W.; Reid, J. P.; Orr-Ewing, A. J. Accurate Measurement of the Optical Properties of Single Aerosol Particles Using Cavity Ring-Down Spectroscopy. *J. Phys. Chem. A* **2022**, *126* (17), 2619-2631. DOI: 10.1021/acs.jpca.2c01246.
(4) Mazurenka, M.; Orr-Ewing, A. J.; Peverall, R.; Ritchie, G. A. D. 4 Cavity ring-down and cavity enhanced spectroscopy using diode lasers. *Annual Reports Section "C" (Physical Chemistry)* **2005**, *101* (0), 100-142, 10.1039/B408909J. DOI: 10.1039/B408909J.
(5) Walker, J. S.; Carruthers, A. E.; Orr-Ewing, A. J.; Reid, J. P. Measurements of Light Extinction by Single Aerosol Particles. *J. Phys. Chem. Lett.* **2013**, *4* (10), 1748-1752. DOI: 10.1021/jz4008068.
(6) Lydersen, A. L.; Greenkorn, R. A.; Hougen, O. A. *Estimation of critical properties of organic compounds by the method of group contibutions*; University of Wisconsin, 1955.
(7) Zuend, A.; Marcolli, C.; Luo, B. P.; Peter, T. A thermodynamic model of mixed organic-inorganic aerosols to predict activity coefficients. *Atmos. Chem. Phys.* **2008**, *8* (16), 4559-4593. DOI: 10.5194/acp-8-4559-2008.
(8) Price, C. L.; Preston, T. C.; Davies, J. F. Hygroscopic Growth, Phase Morphology, and Optical Properties of Model Aqueous Brown Carbon Aerosol. *Environ. Sci. Technol.* **2022**, *56* (7), 3941-3951. DOI: 10.1021/acs.est.1c07356.
(9) CRC handbook of chemistry and physics. *CRC handbook of chemistry and physics.* **1978**.
(10) Lewis, R. J. *Hawley's Condensed Chemical Dictionary*; Wiley, 2007.
(11) Cotterell, M. I.; Willoughby, R. E.; Bzdek, B. R.; Orr-Ewing, A. J.; Reid, J. P. A complete parameterisation of the relative humidity and wavelength dependence of the refractive index of hygroscopic inorganic aerosol particles. *Atmos. Chem. Phys.* **2017**, *17* (16), 9837-9851. DOI: 10.5194/acp-17-9837-2017.
(12) Cornard, J.-P.; Rasmiwetti; Merlin, J.-C. Molecular structure and spectroscopic properties of 4-nitrocatechol at different pH: UV–visible, Raman, DFT and TD-DFT calculations. *Chem. Phys.* **2005**, *309* (2), 239-249. DOI: 10.1016/j.chemphys.2004.09.020.
(13) Djorović, A.; Meyer, M.; Darby, B. L.; Le Ru, E. C. Accurate Modeling of the Polarizability of Dyes for Electromagnetic Calculations. *ACS Omega* **2017**, *2* (5), 1804-1811. DOI: 10.1021/acsomega.7b00171.
(14) Bain, A.; Rafferty, A.; Preston, T. C. The Wavelength-Dependent Complex Refractive Index of Hygroscopic Aerosol Particles and Other Aqueous Media: An Effective Oscillator Model. *Geophys. Res. Lett.* **2019**, *46* (17-18), 10636-10645. DOI: 10.1029/2019GL084568.
(15) Bohren, C. F.; Huffman, D. R. *Absorption and Scattering of Light by Small Particles*; Wiley, 1998.
(16) Hale, G. M.; Querry, M. R. Optical Constants of Water in the 200-nm to 200-µm Wavelength Region. *Appl. Opt.* **1973**, *12* (3), 555-563. DOI: 10.1364/AO.12.000555.
